# TLR4 promotes microglial pyroptosis via lncRNA-F630028O10Rik by activating PI3K/AKT pathway after spinal cord injury

**DOI:** 10.1038/s41419-020-02824-z

**Published:** 2020-08-10

**Authors:** Shun Xu, Jin Wang, Jianyuan Jiang, Jian Song, Wei Zhu, Fan Zhang, Minghao Shao, Haocheng Xu, Xiaosheng Ma, Feizhou Lyu

**Affiliations:** 1 Department of Orthopedics, Shanghai Fifth People’s Hospital, Fudan University, Shanghai, 200240 China; 2 Department of Orthopedics, Huashan Hospital, Fudan University, Shanghai, 200040 China

**Keywords:** Epigenetics, Neuroimmunology

## Abstract

Neuroinflammation plays a crucial role in the secondary phase of spinal cord injury (SCI), and is initiated following the activation of toll-like receptor 4 (TLR4). However, the downstream mechanism remains unknown. Pyroptosis is a form of inflammatory programmed cell death, which is closely involved in neuroinflammation, and it can be regulated by TLR4 according to a recent research. In addition, several studies have shown that long non-coding RNAs (lncRNAs) based mechanisms were related to signal transduction downstream of TLR4 in the regulation of inflammation. Thus, in this study, we want to determine whether TLR4 can regulate pyroptosis after SCI via lncRNAs. Our results showed that TLR4 was activated following SCI and promoted the expression of lncRNA-F630028O10Rik. This lncRNA functioned as a ceRNA for miR-1231-5p/Col1a1 axis and enhanced microglial pyroptosis after SCI by activating the PI3K/AKT pathway. Furthermore, we determined STAT1 was the upstream transcriptional factor of IncRNA-F630028O10Rik and was induced by the damage-responsive TLR4/MyD88 signal. Our findings provide new insights and a novel therapeutic strategy for treating SCI.

## Introduction

Spinal cord injury (SCI) is one of the most serious complications of spinal trauma, and often leads to severe neurological dysfunction due to the irreversible loss of neurons^1^. The repair of damaged spinal tissues is a major clinical challenge due to the complex pathology of acute SCI^2^, which can be divided into the primary injury and secondary injury phases^3^. Primary injury is the initial mechanical insult to the spinal cord, and is followed by secondary injury consisting of decellularization and neuronal necrosis that are caused by changes in the local microenvironment^4^. Studies increasingly show that neuroinflammation is a key factor driving secondary injury post-SCI^5^, and is in turn triggered by inflammasome activation^6^. The inflammasome is a multimeric protein complex comprising of caspase-1, caspase-activating recruitment domain protein (ASC) and sensor NLR proteins (e.g. NLRP1, NLRP2, NLRP3 etc.)^7^. Stimulation of the cytoplasmic inflammasome complex activates caspase-1/4/5/11 and translocates gasdermin (GSDMD)-N domain to the cell membrane, resulting in pore formation and pyroptosis^8^. Microglia, the macrophages of the central nervous system (CNS), are the primary site of neurological pyroptosis^9^, and are therefore promising therapeutic targets for reducing neuroinflammation after SCI.

Toll-like receptor 4 (TLR4) is a key player in innate immune responses that recognizes the pathogen-associated molecular patterns (PAMPs) and damage-associated molecular patterns (DAMPs), and activates the pro-inflammatory nuclear factor kB (NF-κB)^10,11^. Studies show a significant reduction in inflammation and damage-associated mediators in TLR4 deficient mice^12,13^. In addition, TLR4 also regulates GSDMD-mediated pyroptosis in diabetic kidney disease^14^. Besides, in BV-2 cells, Shao et al. found TLR4 phosphorylates NF-κB p65 through PI3K/AKT signaling, accompanied by the activation of downstream pathways and the release of inflammatory factors^15^. In rats with SCI, Dl-3-n-butylphthalide reduces acute glial activation by inhibiting microglial TLR4/NF-κB signaling^16^. Recently, studies have found that TLR4 can regulate inflammation through long non-coding RNAs (lncRNs). Maninjay et al. reported that TLR4 activates NF-κB through MyD88, inhibits the binding of lincRNA-EPS to hnRNPL, maintains the loose state of chromatin, promotes the transcription factor binding to the IRG promoter region, enhances IRG transcription, and promotes inflammatory response^17^. Another research reported TLR4 inhibits the activation of NF-κB and MAPK pathways by inhibiting the ubiquitination rhetoric of ubiquitination ligase TRAF6 by promoting the transcription of lncRNA Mirt2, thereby limiting the production of pro-inflammatory cytokines^18^. Based on the above researches, whether TLR4 can regulate microglial pyroptosis after SCI through lncRNAs has aroused our interest.

LncRNAs are regulatory transcripts that are involved in various pathological processes^19,20^. Several lncNRAs have been identified that are relatively abundant in the CNS and regulate multiple pathways^21,22^. The lncRNAs frequently form competing endogenous RNA (ceRNA) regulatory networks wherein they modulate the expression of downstream genes by competitively binding to microRNAs (miRNAs)^23,24^. CeRNA networks regulating pryoptosis have been reported in different cells types^25,26^, including neurons^27^. In this study, we screened differentially expressed lncRNAs and mRNAs between SCI group and sham-operated group, WT group and TLR4^−/−^ group using RNA sequencing. LncRNA-F630028O10Rik is one of the common differentially expressed lncRNAs, and further RT-PCR verification shows it is the most significantly differentially expressed lncRNA in animals and cells regulated by TLR4. The ceRNA networks prediction found it could regulate PI3K/AKT signaling through lncRNA-F630028O10Rik/miR-1231-5p/Col1a1 axis. And Col1a1 as well as PI3K/AKT pathway are closely related to the inflammatory response according to previous study.

We hypothesized that TLR4 promotes the expression of lncRNA-F630028O10Rik after SCI, which activates the PI3K/AKT pathway through the lncRNA-F630028O10Rik/miR-1231-5p/Col1a1 axis, and finally regulates the microglial pyroptosis after SCI. The aim of this study was to determine the potential roles of TLR4 and ceRNA network(s) in microglial pyroptosis following SCI. We verified a key role of TLR4 in post-SCI neuroinflammation and pyroptosis, and identified the specific ceRNA network. Our findings provide novel insights into the molecular mechanisms underlying SCI, along with a promising therapeutic strategy for nerve repair and regeneration after SCI.

## Results

### TLR4 deficiency enhances motor function recovery after SCI via alleviating microglial pyroptosis

**Fig. 1:**
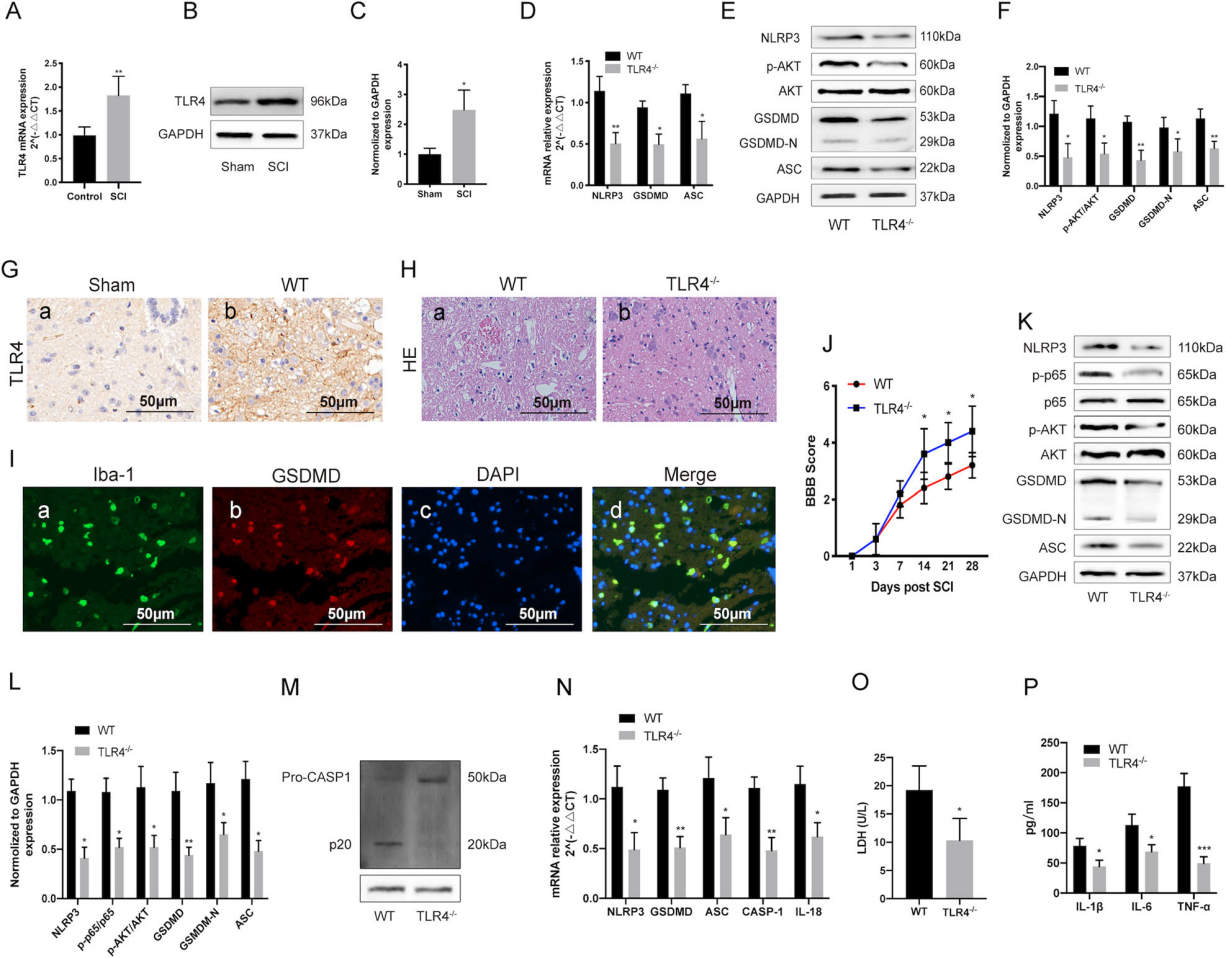
TLR4 deficiency enhances motor function recovery after SCI via alleviating microglial pyroptosis. **a**–**c** TLR4 mRNA and protein levels in the sham-operated and SCI mice 3 days post injury. **d**–**f** The mRNA and protein levels of pyroptosis related genes (NLRP3, GSDMD, GSDMD-N and ASC) and the he phosphorylation of AKT in the injured spinal tissues of WT and TLR4^−/−^ mice 3 days after SCI. **g**, **h** Representative IHC and HE-stained images of the spinal cord of WT and TLR4^−/−^ mice. The immunohistochemical staining was conducted in spinal cord sections at the epicenter and peri-lesion sites. Scale bar: 50 μm. **i** Representative two-photon excitation images of immunofluorescence of Iba-1 and GSDMD acquired from WT or TLR4^−/−^ mice 3 days post-injury or sham surgery. The immunofluorescence staining was conducted in spinal cord sections at the epicenter and peri-lesion sites. **j** BBB scores of WT and TLR4^−/−^ mice on days 1, 3, 7, 14 and 28 after SCI. **k**, **l** Representative Western blotting and statistical comparison of pyroptosis related proteins in LPS-stimulated primary microglia. **m** Representative Western blotting of CASP-1. **n** Relative mRNA expression of pyroptosis related genes in LPS-stimulated primary microglia. **o** The release of LDH was detected by LDH Assay Kit. **p** The release of IL-1β, IL-6 and TNF-α in LPS-stimulated primary microglia. was measured by ELISA. Data are the mean ± SD from 3 independent experiments; *p < 0.05, **p < 0.01.

To determine the potential role of TLR4 in SCI, we first analyzed its expression in the damaged spinal cord tissues of wild type (WT)-SCI mice 3 days post injury, and detected significant elevation in both TLR4 mRNA and protein levels compared to the sham-operated mice (Fig. 1a–c, g). Furthermore, the pyroptosis-related genes including NLRP3, GSDMD and ASC were also upregulated 3 days after SCI, but decreased significantly in the TLR4^−/−^ mice (Fig. 1d–f). Consistent with these results, histological examination of the injured spinal cords showed greater cellular damage in the WT compared to the TLR4^−/−^ mice (Fig. 1h). Immunofluorescence results showed increased expression of GSDMD coincides with Iba-1 (a marker of macrophages/microglia) (Fig. 1i). Furthermore, the BBB scores were also markedly higher among the TLR4^−/−^ mice compared to the WT mice at each time point after SCI (Fig. 1j), indicating better functional recovery in the former. To determine the effect of TLR4 on microglial pyroptosis after SCI in vitro, we generated primary microglia both from TLR4^−/−^ mice and WT mice. We observed that TLR4 deficiency significantly decreased the expression levels of pyroptosis-related genes (NLRP3, ASC, Caspase-1, GSDMD IL-18), inflammatory factors (IL-1β, IL-6 and TNF-α) and LDH compared to the WT group (Fig. 1k, l, n–p). In addition, TLR4 deficiency reduced the phosphorylation of AKT and NF-κB (Fig. 1k) and inhibited the CASP-1 cleavage (Fig. 1m). Taken together, TLR4 is essential for triggering post-SCI pyroptosis, and its deficiency improves motor function recovery after SCI by inhibiting pyroptosis.

### TLR4 upregulates lncRNA-F630028O10Rik after SCI

**Fig. 2:**
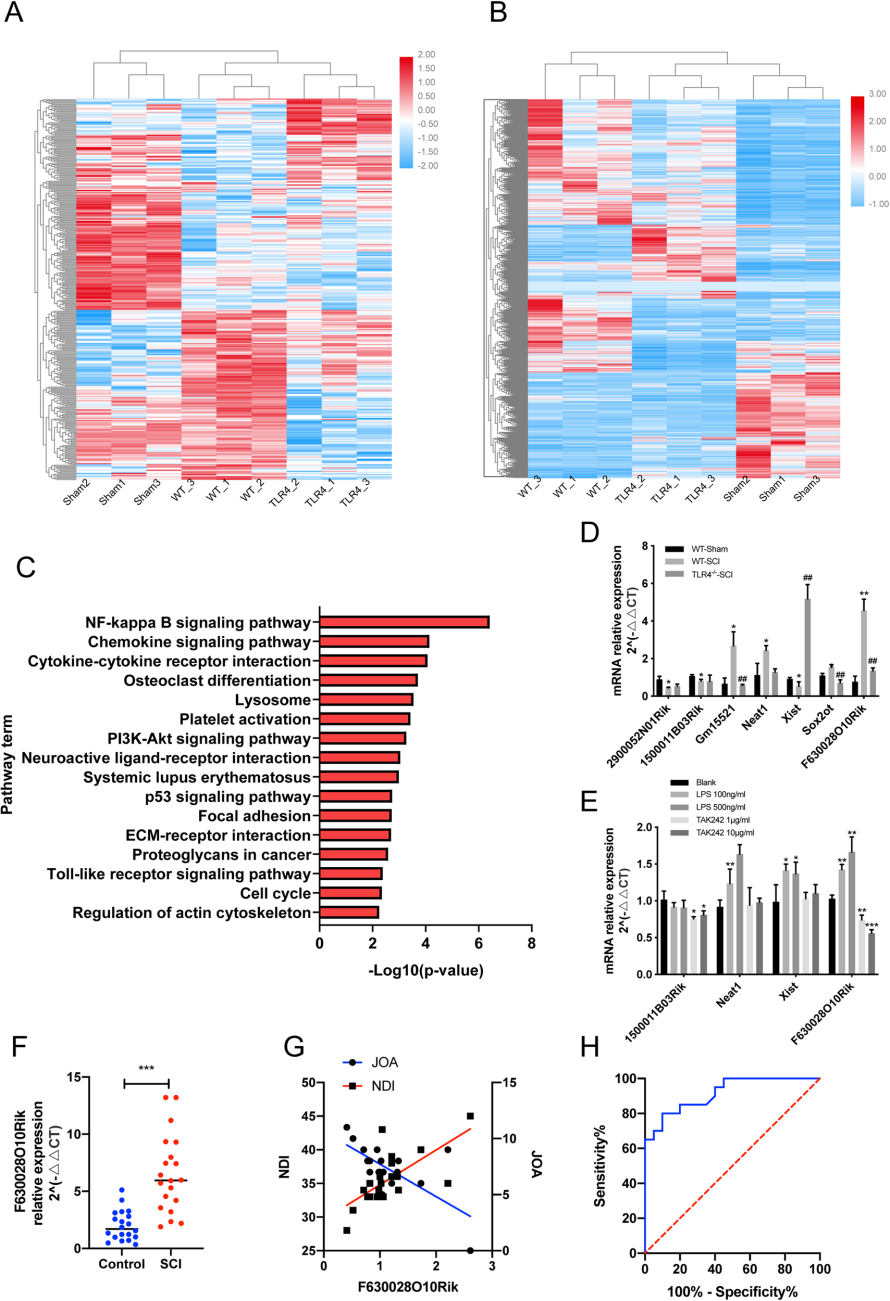
LncRNA-F630028O10Rik was upregulated after SCI and correlated with disease severity. **a**, **b** Heat map showing DELs and DEGs among the WT-SCI, TLR4^−/−^-SCI and the sham-operated mice. **c** KEGG pathway analysis of the DELs. **d** LncRNA levels in the WT-sham, WT-SCI and the TLR4^−/−^-SCI mice. *p < 0.05, **p < 0.01 versus WT-sham; ^##^*p* < 0.01 versus WT-SCI. **e** LncRNA levels in the LPS-stimulated BV2 cells with or without TAK242 treatment. *p < 0.05, **p < 0.01, ***p < 0.001. **f** LncRNA-F630028O10Rik levels in the blood samples of SCI patients and healthy controls. ***p < 0.001. **g** Linear regression analysis of lncRNA-F630028O10Ri expression and JOA scores (*r* = 0.47, *p* < 0.001), and NDI index (*r* = 0.48, *p* < 0.001). **h** The ROC curve analysis of the diagnostic significance of lncRNA-F630028O10Rik (AUC = 0.72) for SCI.

Transcriptomic analysis of the WT sham-operated and SCI mice revealed 621 differentially expressed lncRNAs (DELs) and 2601 differentially expressed genes/mRNAs (DEGs). Among the DELs, 328 lncRNAs were upregulated and 293 were downregulated, whereas 1897 and 704 mRNAs were respectively up- and downregulated. Similarly, 124 lncRNAs and 511 mRNAs were upregulated, and 158 lncRNAs and 899 mRNAs were downregulated between the WT-SCI and TLR4^−/−^-SCI mice (Fig. 2a, b). The ceRNA networks between the DELs and DEGs were then predicted, and KEGG pathway analysis indicated an enrichment in the NF-κB pathway, PI3K/AKT pathway and Toll like receptor pathway (Fig. 2c). LncRNA-F630028O10Rik is one of the common differentially expressed lncRNAs, and further RT-PCR verification shows it is the most significantly differentially expressed lncRNA in animals and cells regulated by TLR4 (Fig. 2d, e). Furthermore, higher levels of lncRNA-F630028O10Rik were detected in the blood samples from SCI patients (positive detection rate was 90% (18/20)) compared to healthy controls (positive detection rate was 85% (17/20)), and also correlated significantly to increased Neck Disability Index (NDI) and decreased Japanese Orthopedic Association (JOA) scores in the patients (Fig. 2f, g). ROC curve analysis further showed high diagnostic accuracy of lncRNA-F630028O10Rik in SCI (Fig. 2h). Interestingly, lncRNA-F630028O10Rik was downregulated in the TLR4^−/−^-SCI mice and in the BV2 cells treated with the TLR4 inhibitor TAK242 (Fig. 2d, e). Taken together, lncRNA- F630028O10Rik is correlated with increased severity of SCI, and its upregulation in the diseased tissues is dependent on TLR4.

### LncRNA-F630028O10Rik abrogates the anti-pyroptotic effect of TLR4 deficiency in microglia after SCI

**Fig. 3:**
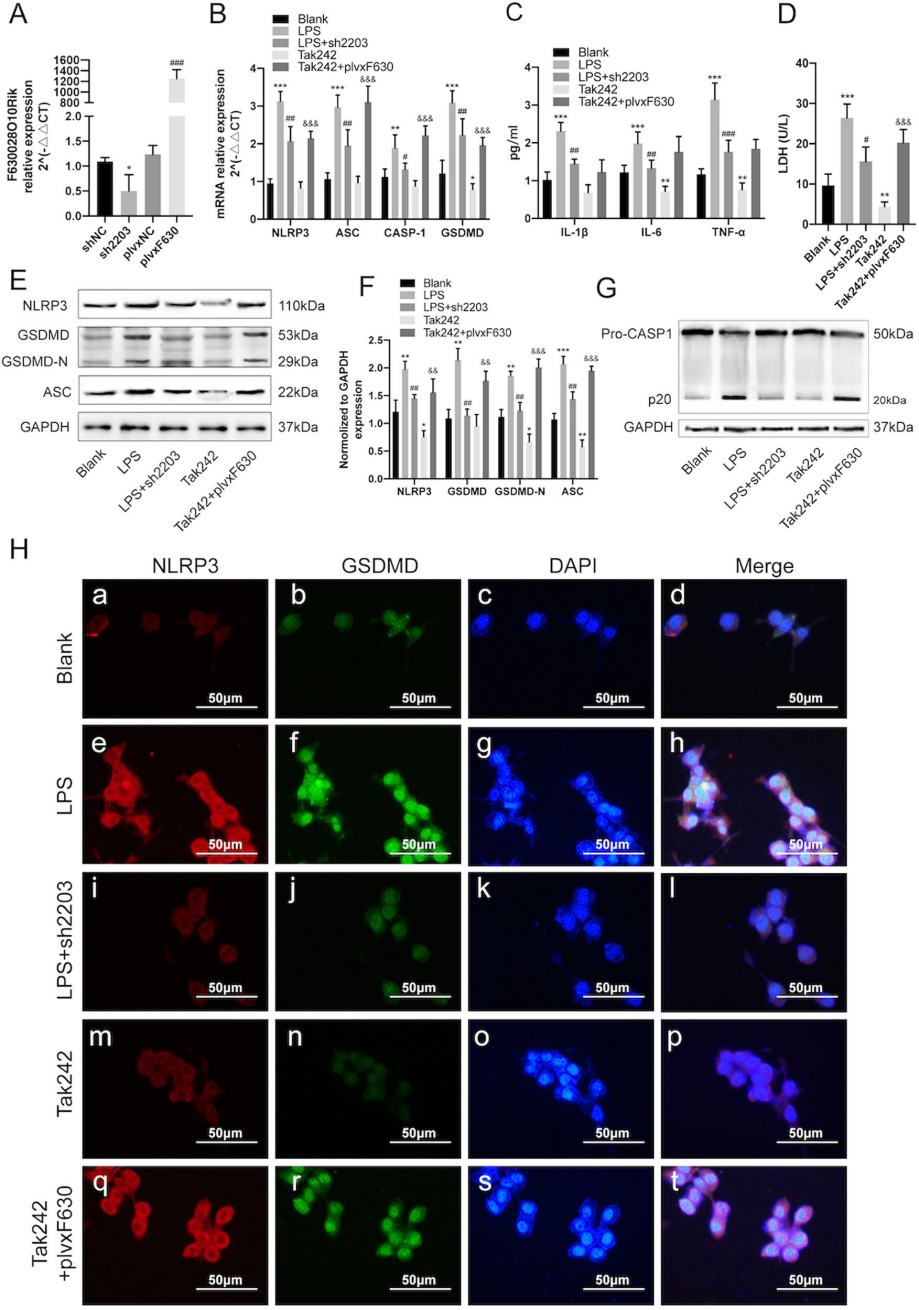
TLR4-induced pyroptosis in vitro relies on lncRNA-F630028O10Rik. **a** LncRNA-F630028O10Rik levels in BV2 cells transfected with sh2203 or plvxF630 constructs. *p < 0.05 versus shNC; ^###^*p* < 0.001 versus plvxNC. **b** NLRP3, ASC, CASP-1 and GSDMD mRNA levels in plvxF630-expressing BV2 cells with/out Tka242 treatment. **c**, **d** IL-1β, IL-6, TNF-a and LDH mRNA levels in Tak242-treated BV2 cells with/out plvxF630 transfection. **e**, **f** Representative immunoblot and quantification of NLRP3, GSDMD, GSDMD-N and ASC proteins in each group. **g** CASP-1 representative immunoblot in Tak242-treated BV2 cells with/out plvxF630 transfection. **h** Representative IHF images showing in situ expression of NLRP3 (Red) and GSDMD (Green) in each group. Nuclei are stained with DAPI (Blue). Scale bar: 50 μm. *p < 0.05, **p < 0.01, ***p < 0.001 versus Blank, ^#^*p* < 0.05, ^##^*p* < 0.01, ^###^*p* < 0.001 versus LPS, ^&&^*p* < 0.01, ^&&&^*p* < 0.001 versus Tak242. Data are the mean ± SD from 3 independent experiments.

**Fig. 4:**
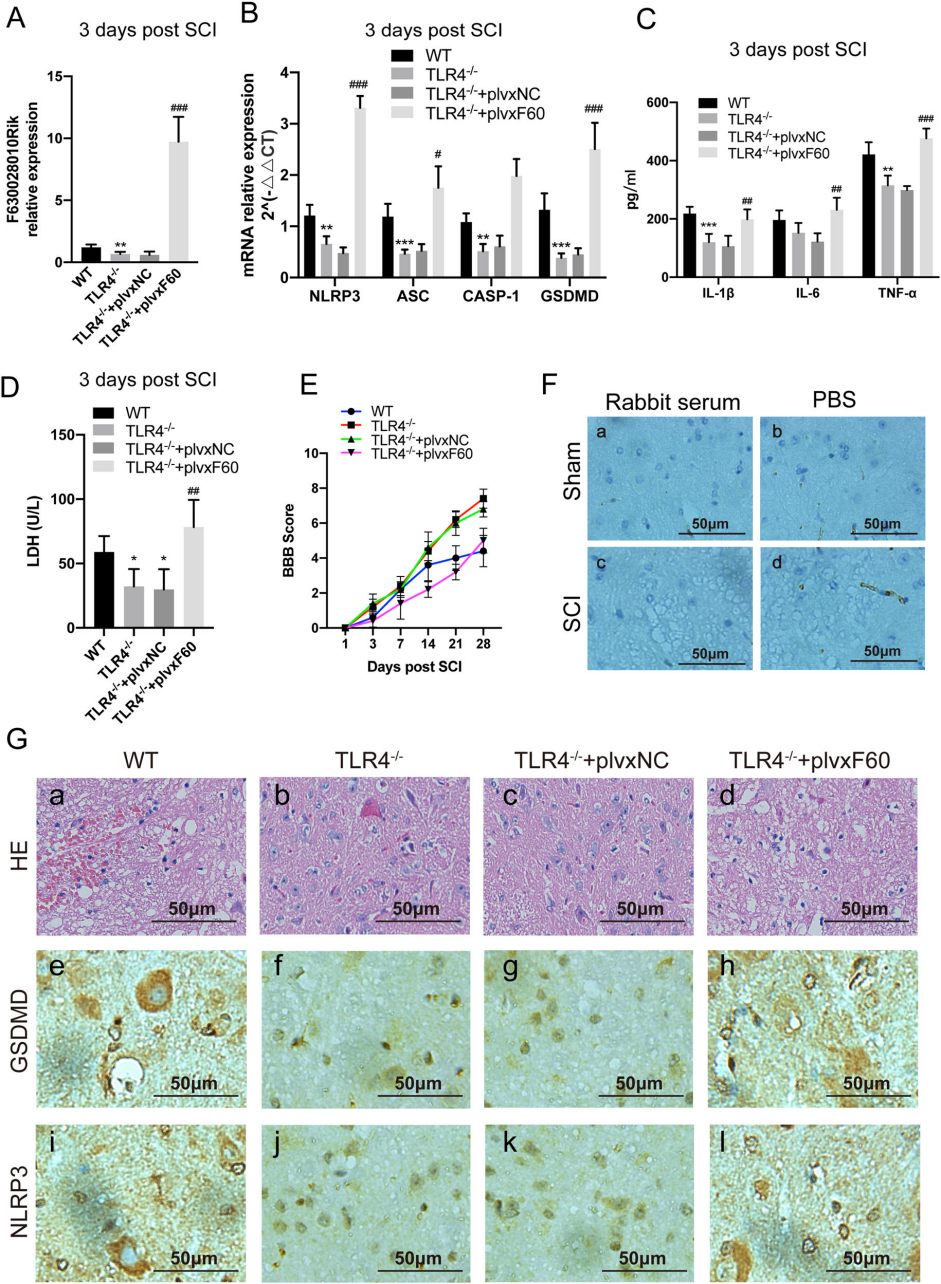
TLR4-induced pyroptosis in vivo relies on lncRNA-F630028O10Rik. **a** LncRNA-F630028O10Rik levels in TLR4^−/−^ mice injected with plvxF60 after SCI. **b** NLRP3, ASC, CASP-1 and GSDMD mRNA levels in TLR4^−/−^ mice with/out plvxF60 treatment. **c**, **d** IL-1b, IL-6, TNF-a and LDH levels in the spinal cord homogenates of the above mice. **e** BBB scores of the different groups at the designated time points. **f** The negative control of IHC. Rabbit serum and PBS were used to instead of primary antibodies to exclude non-specific staining. **g** Representative HE and IHC images of NLRP3 and GSDMD in each group. The immunohistochemical staining was conducted in spinal cord sections at the epicenter and peri-lesion sites. Scale bar: 50 μm. *p < 0.05, **p < 0.01, ***p < 0.001 versus WT, ^#^*p* < 0.05, ^##^*p* < 0.01, ^###^*p* < 0.001 versus TLR4^−/−^+plvxF60.Data are the mean ± SD from 3 independent experiments.

To further determine the role of lncRNA-F630028O10Rik in microglial pyroptosis, we generated stable BV2 lines with lncRNA-F630028O10Rik knockdown or overexpression. The mus2203 siRNA sequence resulted in optimum silencing of lncRNA-F630028O10Rik (Supplementary Fig. 1a), and significantly decreased the expression levels of pyroptosis-related genes (NLRP3, ASC, Caspase-1, GSDMD), inflammatory factors (IL-1β, IL-6 and TNF-α), LDH compared to the siNC group (Supplementary Fig. 1b–g). In addition, the above markers and CASPA-1 cleavage were significantly downregulated in the LPS-stimulated cells additionally transfected with sh2203 or treated with Tak242 compared to the LPS-stimulated controls (Fig. 3a–g). Consistent with this, overexpression of lncRNA-F630028O10Rik in BV2 cells elevated the inflammasome-related factors and promoted pyroptosis, even upon pharmacological TLR4 inhibition (Fig. 3a–h). Furthermore, ectopic expression of lncRNA630028O10Rik in the TLR4^−/−^ mice nullified the anti-pyroptotic effects of TLR4 deficiency (Fig. 4a–d, g), as well as the improvements in BBB scores (Fig. 4e). Thus, lncRNA-F630028O10Rik mediates TLR4-induced pyroptosis after SCI, and can also promote the pyroptotic pathway independent of TLR4.

### LncRNA-F630028O10Rik sponges miR-1231-5p to upregulate Col1a1 after SCI

**Fig. 5:**
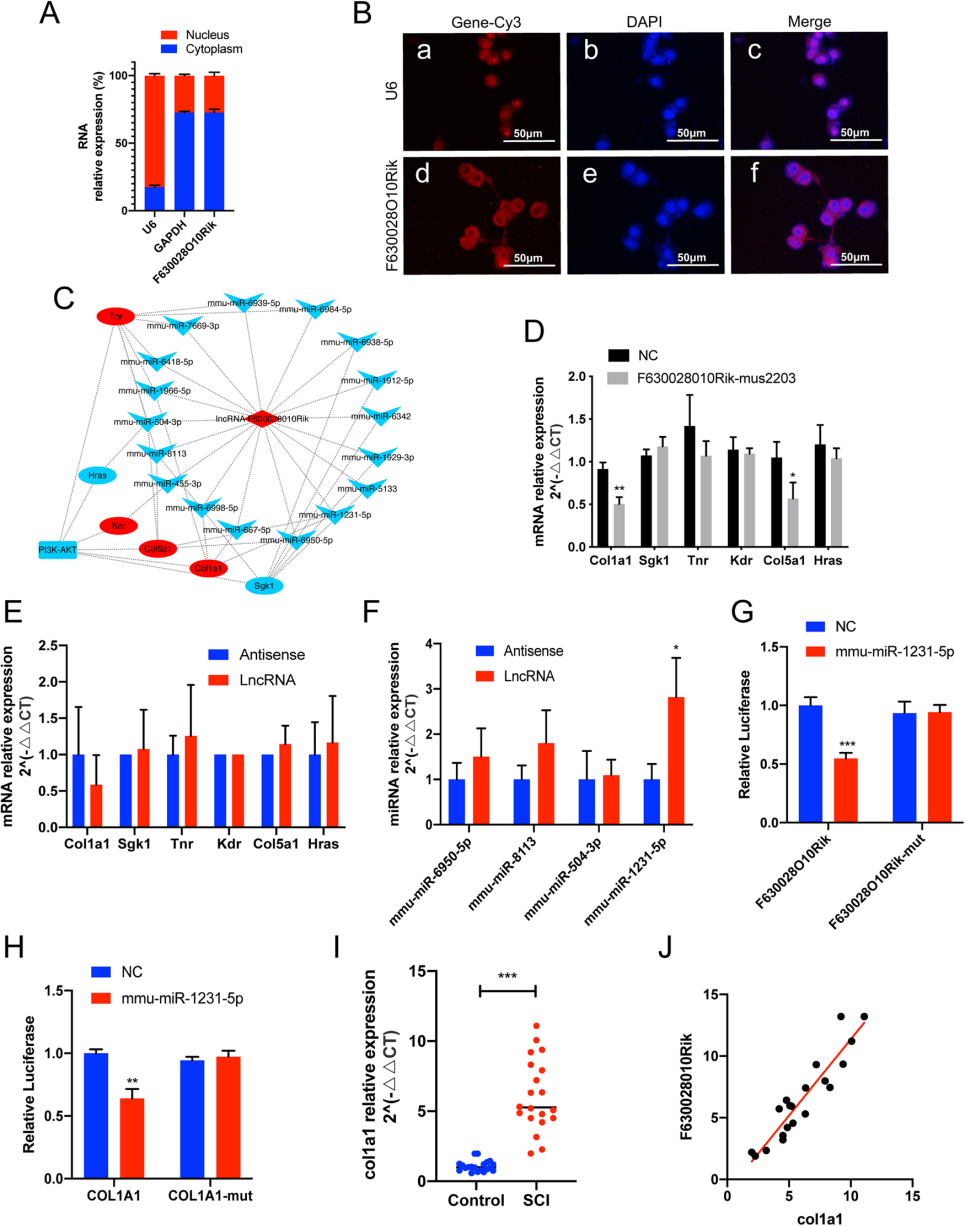
LncRNA-F630028O10Rik acts as a sponge for miRNA-1231-5p to regulate Col1a1. **a** The relative expression of nuclear and cytosolic lncRNA-F630028O10Rik. **b** RNA-FISH images showing the subcellular localization of lncRNA-F630028O10Rik in BV2 cells. Scale bar: 50 μm. **c** CeRNA network predicted by miRanda (http://www.microrna.org/microrna/home.do) and RNAhybrid (https://bibiserv.cebitec.uni-bielefeld.de/rnahybrid). Red diamonds represent lncRNAs, red ellipses represent mRNAs, and blue V-shapes represent miRNAs. **d** The expression of predicted target genes in NC and lncRNA-F630028O10Rik-mus2203 cells. **e**, **f** RNA pull-down assay showing the physical relationship between lncRNA-F630028O10Rik and the predicted miRNAs (**f**) or target genes (**e**). **g**, **h** Relative luciferase activity of lncRNA-F630028O10Rik (**g**) and Col1a1 (**h**) promoter-driven reporter constructs. **i** The relative expression of Col1a1 in the blood samples of SCI patients and healthy controls. **j** Linear regression between lncRNA-F630028O10Rik and Col1a1 (*r* = 0.87, *p* < 0.001). *p < 0.05, **p < 0.01, ***p < 0.001. Data are the mean ± SD from 3 independent experiments.

**Fig. 6:**
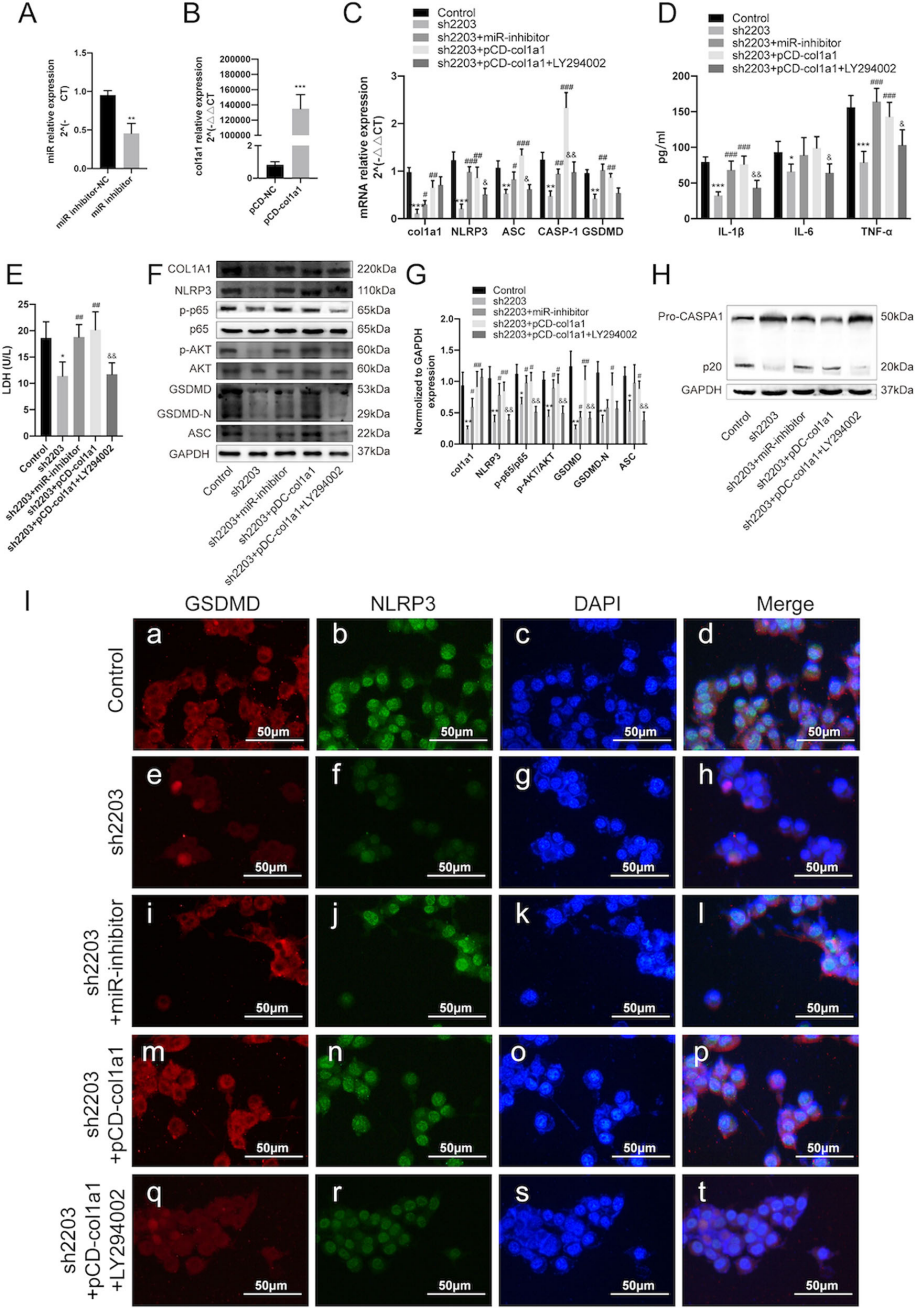
LncRNA-F630028O10Rik/miR-1231-5p/Col1a1 mediated pyroptosis via the PI3K/AKT pathway. **a** MiR-1231-5p was downregulated by the specific inhibitor. **p < 0.01. **b** Col1a1 was upregulated by pCD-Col1a1. ***p < 0.001. **c** Expression levels of Col1a1 and pyroptosis-related genes in cells transfected with miR-inhibitor, pCD-Col1a1 and sh2203, with/out LY294002 treatment. **d**, **e** Expression levels of inflammatory kinases and LDH in cells transfected with miR-inhibitor, pCD-Col1a1 and sh2203, with/out LY294002 treatment. **f**, **g** Representative immunoblot and quantification of the indicated proteins in each group. **h** CASP-1 representative immunoblot in each group. **i** Representative immunofluorescence images showing in situ expression of GSDMD (Red) and NLRP3 (Green) in each group. Nuclei are stained with DAPI (Blue). *p < 0.05, **p < 0.01, ***p < 0.001 versus Control, ^#^*p* < 0.05, ^##^*p* < 0.01, ^###^*p* < 0.001 versus sh2203, ^&^*p* < 0.05, ^&&^*p* < 0.01 versus sh2203+pCD-col1a1. Data are the mean ± SD from 3 independent experiments.

As shown in Fig. 5a, b, lncRNA-F630028O10Rik was mainly localized in the cytosol, strongly indicating its involvement in a ceRNA network. Therefore, we next predicted the ceRNA networks targeting the PI3K-AKT signaling pathway using miRanda and RNAhybrid programs (Fig. 5c), and verified Col1a1 and Col5a1 as the target genes that were downregulated in lncRNA-F630028O10Rik-knockdown cells (Fig. 5d). RNA pulldown assay further showed that the lncRNA bound to miR-1231-5p rather than the target mRNAs (Fig. 5e, f). The relationship between miR-1231-5p and lncRNA-F630028O10Rik was then validated by the luciferase reporter gene assay using WT or mutant 3’-UTR sequence of lncRNA-F630028O10Rik. As shown in Fig. 6g, miR-1231-5p significantly inhibited the activity of luciferase reporter controlled by the WT but not mutant lncRNA-F630028O10Rik promoter (Fig. 5g). Furthermore, miR-1231-5 also suppressed the luciferase activity of reporter gene under Col1a1 promoter (Fig. 5h). Thus, the 3’-UTR sequences of both lncRNA-F630028O10Rik and Col1a1 are the direct targets of miR-1231-5p. Finally, Col1a1 levels were significantly increased in SCI patients and positively correlated with that of lncRNA-F630028O10Rik (Fig. 5i, j). Taken together, lncRNA-F630028O10Rik acts as a ceRNA for miR-1231-5p in SCI, and sponges the latter to upregulate Col1a1.

### LncRNA-F630028O10Rik/miR-1231-5p/Col1a1 regulates post-SCI pyroptosis via the PI3K/AKT pathway

The potential role of the lncRNA-F630028O10Rik/miR-1231-5p/Col1a1 axis in pyroptosis was further analyzed by gain/loss functional experiments using miR-1231-5p inhibitor (Fig. 6a) and pCD-Col1a1 (Fig. 6b). The miR-1231-5p inhibitor restored Col1a1 expression in the lncRNA-F630028O10Rik-sh2203 cells (Fig. 6c). Furthermore, the inflammatory cytokines, LDH, pyroptosis-related genes and CASP-1 activation were also upregulated in the sh2203-transfected cells following miR-1231-5p inhibition and Col1a1 overexpression (Fig. 6c–h). Finally, the pro-pyroptotic effects and p65 activation were abolished when the PI3K/AKT signaling pathway was specifically inhibited by LY294002 (Fig. 6c–i). Taken together, the lncRNA-F630028O10Rik/miR-1231-5p/Col1a1 axis regulates pyroptosis via the PI3K/AKT signaling pathway.

### TLR4/MyD88 signaling regulates lncRNA-F630028O10Rik through STAT1

**Fig. 7:**
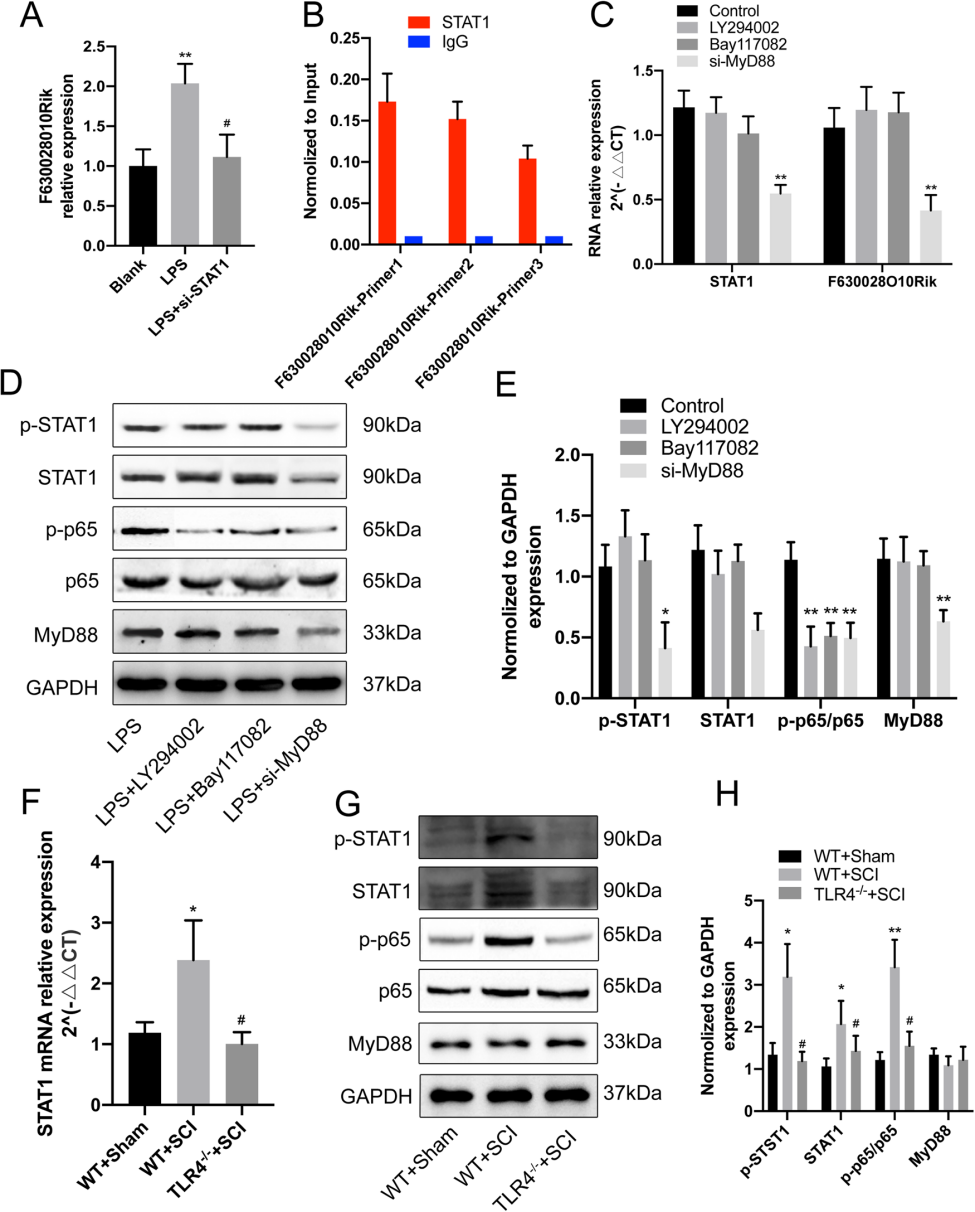
TLR4/MyD88/STAT1 upregulated lncRNA-F630028O10Rik. **a** LncRNA-F630028O10Rik levels in LPS-treated cells following STAT1 silencing. **p < 0.01 versus Blank, ^#^*p* < 0.05 versus LPS. **b** ChIP assay showing binding between STAT1 and lncRNA-F630028O10Rik. **c** LncRNA-F630028O10Rik and STAT1 levels in MyD88-knockdown cells. **p < 0.01. **d**, **e** Representative immunoblot and quantification of p-STAT1, STAT1, p-p65, p65 and MyD88 in each group. *p < 0.05, **p < 0.01. **f** STAT1 mRNA levels in WT-Sham, WT-SCI and TLR4^−/−^-SCI mice. *p < 0.05 versus WT-Sham, ^#^*p* < 0.05 versus WT+SCI. **g**, **h** Representative immunoblot and quantification of p-STAT1, STAT1, p-p65, p65 and MyD88 in WT-Sham, WT-SCI and TLR4^−/−^-SCI mice. *p < 0.05 versus WT-Sham, ^#^*p*< 0.05 versus WT+SCI. Data are the mean ± SD from 3 independent experiments.

The upstream regulator of lncRNA-F630028O10Rik was next identified by screening for putative transcriptional factors (TFs) in the JASPAR database, and STAT1 was one of the predicted TFs. Indeed, siRNA-mediated silencing of STAT1 markedly downregulated lncRNA-F630028O10Rik in the LPS-stimulated cells (Fig. 7a). ChIP assay further showed direct binding between STAT1 and lncRNA-F630028O10Rik (Fig. 7b). In addition, STAT1 and lncRNA-F630028O10Rik levels were not significantly affected after blocking the PI3K/AKT or NF-κB signaling pathways (Fig. 7c–e). In contrast, silencing MyD88, the intracellular adaptor of TLR4, significantly downregulated STAT1 mRNA and lncRNA-F630028O10Rik, as well as p-STAT1 levels (Fig. 7c–e). In addition, inhibition of PI3K/AKT signaling or silencing MyD88 reduced the activation of NF-κB (Fig. 7d, e, g, h). Consistent with this, STAT1 expression and NF-κB activation were significantly higher in the WT-SCI mice compared to the WT-Sham mice, and reversed in the TLR4^−/−^-SCI mice (Fig. 7f–h). In conclusion, STAT1 is the upstream regulator of lncRNA-F630028O10Rik and is in turn regulated via TLR4/MyD88 signaling.

## Discussion

SCI is a debilitating condition with life-threatening complications, and more than 250,000 people suffer from acute SCI annually as per WHO statistics^28^. However, there is still no effective therapy against SCI at present due to its complex pathological mechanism. Aberrant neuroinflammation is a key factor in CNS trauma, and its inhibition is a highly promising therapeutic strategy^29–31^. The inflammasome complex is activated after SCI^32^, and is known to trigger pyroptosis in various CNS traumas. However, the causative role of pyroptosis in SCI, and the possible involvement of TLR4 in CNS inflammation remains to be established^33,34^. We found that TLR4 was markedly upregulated in the injured spinal cords, and its deficiency attenuated pyroptosis in the SCI mice by downregulating NLRP3, GSDMD and ASC, and accelerated locomotor recovery. These results are in line with previous reports stating that downregulation of TLR4 alleviates inflammation and promotes recovery after SCI^35,36^.

LncRNAs regulate the inflammatory response in various pathological conditions^37,38^. For example, Zhang et al. reported an anti-inflammatory role of lncRNA-MALAT1 in ischemic stroke^39^. In addition, MALAT1 inhibits neuronal apoptosis and neuroinflammation, and stimulates neurite outgrowth in Alzheimer’s disease^40^. Zhong et al. observed that the upregulation of lncRNA-Neat1 alleviated neuroinflammation in mice after traumatic brain injury, and improved functional recovery^41^. In line with these findings, the lncRNA-F630028O10Rik was markedly upregulated following SCI, and decreased in the TLR4^−/−^ mice. In addition, lncRNA-F630028O10Rik expression correlated with worse locomotor function after SCI and thus could be regarded as a novel diagnostic index for SCI.

There is considerable evidence supporting increased microglial pyroptosis in CNS diseases^42,43^. Furthermore, several lncRNAs have been identified that regulate the function of macrophages/microglia. For example, Zhang et al. found that lncRNA-1810034E14RiK inhibited microglia activation in experimental ischemic stroke^44^, and Sun et al. reported that lncRNA-GAS5 inhibited microglial M2 polarization and exacerbated demyelination^45^. In our study, ectopic expression of lncRNA-F630028O10Rik in the murine microglial BV2 cells significantly increased the expression levels of pyroptosis and inflammation-related genes. Interestingly, TLF4 deficiency downregulated lncRNA-F630028O10Rik as well as the downstream factors both in vivo and in vitro, while forced expression of lncRNA-F630028O10Rik exacerbated pyroptosis following pharmacological inhibition of TLR4. Thus, lncRNA-F630028O10Rik is an essential mediator of TLR4-induced pyroptosis after SCI. LncRNAs regulate gene expression indirectly by targeting miRNAs through various mechanisms. They can act as miRNA sponges (also known as ceRNAs), compete with miRNAs for the target mRNAs, be processed into miRNAs, and transport the miRNAs to target mRNAs^46–48^. Various ceRNAs have been identified in CNS injuries. For example, Han et al. reported that lncRNA-MEG3 decreased neural cell apoptosis after hypoxic damage by targeting miR-147^49^. Similarly, we identified the lncRNA-F630028O10Rik/miR-1231-5p/Col1a1 ceRNA network that regulated pyroptosis in microglia.

Collagen is a major extracellular matrix protein^50^, and type I collagen in particular acts as a signaling molecule in inflammation. Zhang et al. reported that collagen I stimulated recruitment and aggregation of mouse peritoneal macrophages, as well as the production of pro-inflammatory cytokines by increasing ROS levels^51^. Molokanova et al. found that collagen type I deficiency (but not complete absence) attenuated inflammatory cell activation/recruitment in the damaged liver^52^. We detected significantly higher levels of Col1a1 in the blood samples of SCI patients, further underscoring its involvement in post-SCI neuroinflammation. The PI3K/AKT pathway regulates multiple physiological and pathological processes, including inflammation. Activation of the TLR4/PI3K/AKT/GSK3β signaling pathway in macrophages/microglia aggravated neuroinflammation in Parkinson’s disease. Furthermore, LPS-induced lung injury was also mediated via this pathway^53,54^. We found that the AKT inhibitor alleviated pyroptosis in the microglia, thus confirming that the lncRNA-F630028O10Rik/miR-1231-5p/Col1a1 axis regulated microglial pyroptosis via the PI3K/AKT pathway.

LncRNAs are regulated by various upstream TFs^55,56^. JASPAR database prediction and ChIP assay identified signal transducer and activator of transcription 1 (STAT1) as the upstream regulator of lncRNA-F630028O10Rik. The TLRs relay signals through cytosolic Toll/IL receptor domain adapter proteins, such as Mal, TRIF, TRAM and MyD88, resulting in the phosphorylation of several TFs including STAT1 and NF-κB^57^. Consistent with this, MyD88 silencing significantly downregulated STAT1, which further strengthens the link between TLR4 signaling and induction of pyroptosis after SCI.

The NF-κB family is the most extensively studied transcription factor due its critical role in inflammation related responses^58,59^. They can form homo- and heterodimers via N-terminal DNA-binding domain and bind to a various of related target DNA sequences called κB sites to modulate gene expression^59^. TLR4 is a key player in innate immune responses that recognizes PAMPs (mainly LPS), activating the NF-κB pathway which may the most important signal to be involved in the activation of NLRP3 inflammasome and pyroptosis, and we really found TLR4 deficiency significantly decreased the activation of p65 in mice after SCI. In addition, researchers have found TLR4 phosphorylates NF-κB p65 through PI3K/AKT signaling. To determine the relationship between PI3K/AKT and NF-κB in pyroptosis of microglia after SCI, we detected the phosphorylation of NF-κB p65 in BV2 cells after treated with PI3K inhibitor LY294002. The results showed the activation of p65 is suppressed after PI3K/AKT pathway is blocked. Therefore, we believe that in microglia, TLR4 promotes PI3K activation through lncRNA-F630028O10Rik, which in turn affects the activation of NF-κB p65 and has an effect on microglial pyroptosis. This pathway may be an important complement to traditional pathways at the epigenetic level in microglia.

**Fig. 8:**
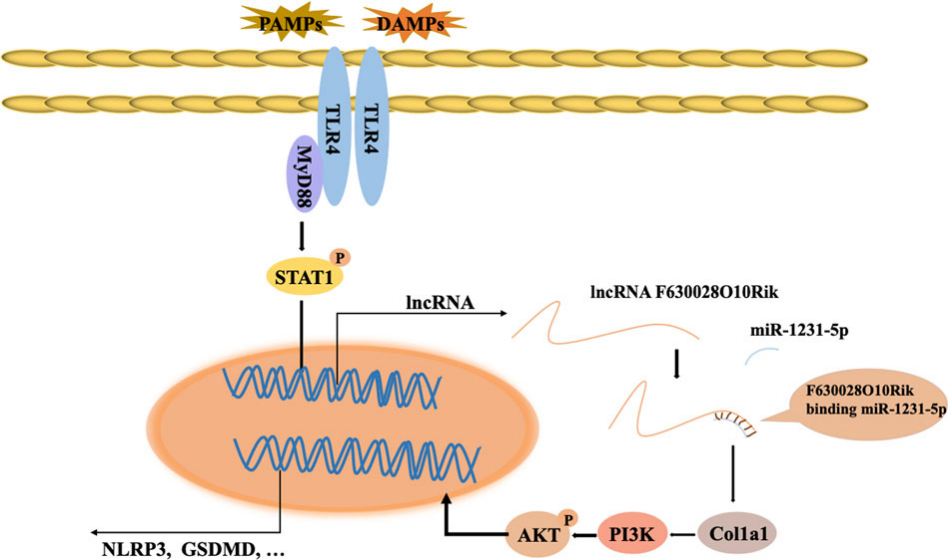
The mechanism of post-SCI microglial pyroptosis. TLR4/MyD88 relays damage signals to macrophage/microglia to promote the phosphorylation of STAT1 and upregulate lncRNA-F630028O10Rik. The latter acts as the sponge for miR-1231-5p to upregulate Col1a1, leading to increased pyroptosis via the PI3K/AKT signaling pathway.

In conclusion, TLR4/MyD88 relays the damage signals following SCI into the macrophages/microglia, which phosphorylates STAT1 and activates the downstream lncRNA-F630028O10Rik/miR-1231-5p/Col1a1 ceRNA network that triggers pyroptosis via the PI3K/AKT pathway (Fig. 8). This novel regulatory axis is a promising therapeutic target against SCI.

## Materials and methods

### Human blood samples

Twenty patients diagnosed with SCI at the Orthopedics Department of Huashan Hospital, Fudan University, and 20 age- and sex-matched healthy donors were enrolled. The severity of SCI was assessed by Japanese orthopedic association (JOA) scores and cervical dysfunction index (NDI). Peripheral blood was collected from all participants. The clinical characteristics of patient samples were showed in Supplementary Table 1. The Ethics Committee of Fudan University Huashan Hospital approved the study protocol, and informed consent was obtained from each donor.

### Establishment of SCI model

The wild-type (WT) and TLR4 knock out (KO) C57BL/6 male mice were purchased from Animal center of the Chinese Academy of Science (Shanghai, China), and housed in a humidity and temperature-controlled environment with free access to food and water. All animal experiments were approved by the Institutional Animal Care and Use Committee of Fudan University. The SCI model was established as previously described^29^. Briefly, the mice were weighed and anesthetized with 35 mg/kg 4% pentobarbital via intraperitoneal injection. The T8-T9 vertebrae were exposed by laminectomy using a pair of fine rongeurs, and the dura mater was protected. Spinal injury was inflicted by laterally compressing the cord with Dumont-type forceps to a depth of 0.2 mm for 20 s. The incision was sutured layer by layer and 20,000 units penicillin was injected intramuscularly once daily for 3 days. Bladder was emptied manually 3 times a day. The mice were injected intraperitoneally with plvxNC or plvxF60 (5 × 10^7^ IU) 1 day before surgery as well as every day after establishing the SCI model as per the experimental requirements.

### Assessment for locomotor function

The locomotor function was scored using the Basso, Beattie and Bresnahan (BBB) scale at 0, 3, 7, 14, 21 and 28 days after SCI from 0 (no observable movement) to 21 points (normal movement with parallel paw placement). The mice were placed in an open field and allowed to move freely for 5 minutes, and the hindlimb movements, stepping and coordination points were assessed by an investigator who was blinded to grouping.

### Cell culture and transfection

Mixed glial cell cultures were generated from the cerebral cortex of postnatal (24 h old) mice and cultured in Dulbecco’s modified Eagle’s medium/F12 containing 10% fetal bovine serum (FBS; Gibco, Carlsbad, CA, USA) and an antibiotic mixture (1% penicillin/streptomycin) (Invitrogen, Carlsbad, CA, USA) at 37 °C and 5% CO_2_ for 10 days. Cultures were shaken for 6 h at 180 rpm at 37 °C to collect and purify microglia. BV2 cells were cultured in DMEM (Gibco, Carlsbad, CA, USA) supplemented with 10% FBS (Gibco, Carlsbad, CA, USA), 50 g/ml streptomycin (Invitrogen, Carlsbad, CA, USA) and 50 U/ml penicillin in a humidified atmosphere containing 5% CO_2_. The siRNAs, shRNAs, plasmids and lentiviral constructs (Supplementary Data 1) were purchased from Genechem Co. Ltd. (Shanghai, China), and siRNA-MyD88 was purchased from ThermoFisher (Catalog #: AM16708, CA, USA). The BV2 cells were transfected with the different constructs using Lipofectamine™ 3000 reagent (Invitrogen, Carlsbad CA, USA) according to the manufacturer’s instructions. For lentiviral transduction, the cells were incubated with 100 μl lentivirus particles and 5 μg/ml polyberence in 2 ml medium in a 6-well plate for 24 h. The miRNA inhibitor was transfected at the final concentration of 100 nM using the riboFECTTM CP Kit (Ribobio, Shanghai, China) according to the manufacturer’s instructions. In addition, the cells were treated with different combinations of LPS (1 μg/ml), TAK242 (10 μg/ml), LY294002 (50 μM) and BAY117082 (5 mM) with/without transfection. The cells were harvested 8 h later, and mRNA and protein were isolated.

### Enzyme-linked immunosorbent assay

IL-1β, IL-6 and TNF-α levels in cell culture supernatants and mouse spinal cord homogenates were analyzed using commercial ELISA kits from Sigma (Sigma-Aldrich, St. Louis, MO, USA) according to the manufacturer’s instructions.

### Cytotoxicity assay

The secreted levels of lactate dehydrogenase (LDH) was detected using the LDH Cytotoxicity Assay Kit (Beyotime, Shanghai, China) according to the manufacturer’s instructions.

### Quantitative real-time PCR (RT-PCR)

RNA was extracted from whole blood using GeneJET Stabilized and Fresh Whole Blood RNA Kit (ThermoFisher, CA, USA), and from the harvested BV2 cells using Trizol (Invitrogen, Carlsbad, CA, USA) according to the manufacturers’ instructions. The total RNA was reverse-transcribed, and the cDNAs were amplified by 40 cycles of denaturation at 95 °C for 1 min, annealing at 55 °C for 1 min and extension at 72 °C, and final 5-min extension at 72 °C. GAPDH was used as the housekeeping gene and relative expression levels of the mRNAs were calculated by the comparative ΔΔCT method. Primers sequences are shown in Supplementary Table 2.

### RNA sequencing and functional enrichment analysis

Total RNA was isolated from spinal cord tissues 3 days post SCI using Trizol (Invitrogen, Carlsbad CA, USA) according to the manufacturer’s protocol. RNA integrity was evaluated using the Agilent 2200 TapeStation (Agilent Technologies, USA). Purified RNAs with RIN score > 7 were reverse transcribed, followed by adaptor ligation and low cycle enrichment using NEBNext^®^ Ultra™ RNA Library Prep Kit for Illumina (NEB, USA). The purified l library products were evaluated using the Agilent 2200 TapeStation and Qubit^®^2.0 (Life Technologies, USA) and then diluted to 10pM for cluster generation *in situ* on the pair-end flow cell, followed by sequencing (2 × 150 bp) on HiSeq3000. The clean reads were obtained after removing low quality reads, and those containing adapter and ploy-N sequences. HISAT2 was used to align the clean reads to the mouse reference genome mm10 with default parameters, and then converted into read counts for each gene model using HTseq. Differential expression was assessed by DEseq using read counts as the input, with Benjamini–Hochberg multiple test correction. The differentially expressed genes (DEGs) were selected based on fold change > 2 and adjusted *p*-value < 0.05 as the thresholds. The ceRNA networks were predicted using miRanda (http://www.microrna.org/microrna/home.do) and RNAhybrid (https://bibiserv.cebitec.uni-bielefeld.de/rnahybrid).

### Western blotting

Protein was extracted from the spinal cord tissues and BV2 cells using RAPI lysis buffer, and quantified by the BCA assay. Protein samples were separated by sodium dodecyl sulfate-polyacrylamide gel electrophoresis (SDS-PAGE), and then transferred to nitrocellulose membranes. After blocking with 5% skimmed milk for 1 h, the membranes were incubated overnight with antibodies against TLR4 (1:500, Abcam, ab13556), NLRP3 (1:1000, Abcam, ab214185), CASP-1 (1:1000, Abcam, ab207802), p65 (1:1000, CST, 8242), p-p65 (1:1000, CST, 3033), STAT1 (1:1000, Abcam, ab3987), p-STAS1 (1:1000, Abcam, ab109461), AKT (1:1000, CST, 2920), p-AKT (1:1000, CST, 4070), GSDMD (1:1000, Abcam, ab210070), ASC (1:1000, CST, 67824), MyD88 (1:1000, Abcam, ab2064), Col1a1(1:1000, CST, 84336) and GAPDH (1:2000, Abcam, ab8245) at 4 °C. The membranes were washed with Tris-buffered saline containing 0.1% Tween-20 (TBST), and then incubated with HRP-conjugated secondary antibody at room temperature for 1 h. The protein bands were detected using an enhanced chemiluminescence (ECL) kit, and quantified by the gel imaging system (UVP LLC, Upland, CA, USA).

### Immunohistochemistry

On the third day after SCI, the mice were deeply anesthetized with 10% chloralic hydras (3.5 ml/kg, i.p.) and perfused with 0.9% NaCl. The spinal cord segments near the lesion epicenter were resected, fixed overnight with 4% paraformaldehyde, and embedded in paraffin. Three transverse 25µm-thick sections were mounted on poly-L-lysine-coated slides. For immunohistochemical staining, deparaffinized sections were incubated with H_2_O_2_ and methanol for 10 min to block endogenous peroxidase, and then with serum-blocking solution for 30 min. The sections were then incubated with primary antibodies against TLR4 (1:100, Abcam, ab13556), GSDMD (1:100, Abcam, ab210070) and NLRP3 (1:100, Abcam, ab214185) for 1 h, followed by HRP-conjugated anti-rabbit secondary antibodies for 30 min. Color was developed with the peroxidase substrate DAB for 10 min at room temperature, and the slides were observed under Nikon ECLIPSE Ti microscope (Nikon, Japan). Rabbit serum and PBS were used to instead of primary antibodies to exclude non-specific staining and the results were showed in Supplementary Fig. 4.

### Immunofluorescence

Suitably treated BV2 cells were fixed with 4% paraformaldehyde in 0.1 M phosphate buffer (pH 7.4) for 15 min, and blocked for 1 h with 1% bovine serum albumin containing 0.3% Triton X-100. Following overnight incubation with anti-GSDMD (1:100, Abcam, ab210070) and anti-NLRP3 (1:100, Abcam, ab1872) primary antibodies at 4 °C, the samples were incubated for 2 h at room temperature with Dylight (Dy)488- and Dy594-conjugated secondary antibodies (all 1:1000; Jackson ImmunoResearch, West Grove, PA). All samples were imaged with Nikon ECLIPSE Ti microscope (Nikon, Japan).

### Cytoplasmic and nuclear RNA fractionation

Nuclear and Cytoplasmic Extraction Reagents (ThermoFisher, CA, USA) were used for nuclear-cytoplasm separation, and RNA was extracted for RT-PCR. GAPDH and U6 were used as the respective positive controls for cytoplasmic and nuclear RNA.

### RNA-FISH

BV2 cells were incubated with digoxin-labeled probe sequences targeting lncRNA-F630028O10Rik using the Fluorescent in Situ Hybridization Kit (RiboBio, Shanghai, China) according to the manufacturer’s directions.

### RNA pull-down

BV2 cells were transfected with biotinylated lncRNA-F630028O10Rik or lncRNA-F630028O10Rik antisense sequences (Supplementary Data 2). The Pierce Magnetic RNA-Protein Pull-Down Kit (ThermoFisher, MA, USA) was used for RNA pull-down assay. Briefly, the biotinylated lncRNAs were captured with streptavidin magnetic beads and incubated with the cell lysates at 4 °C for 6 h. The mixture was washed and eluted, and the eluent was analyzed by RT-PCR.

### Dual-luciferase reporter assay

Luciferase reporters were generated by cloning lncRNA-F630028O10Rik and Col1a1 into pisCHECK vector (Sequence are showed in Supplementary Data 3). The 293 T cells were transfected with 100 ng reporter and 100 nM mmu-miR-1231-5p per well using Lipofectamine™ 3000 reagent (Invitrogen, Carlsbad CA, USA). The medium was replaced after 8 h with the complete culture medium. The cells were harvested 48 h after transfection, and luciferase activity was measured using toolVeritas 9100-002 (Turner BioSystems, Sunnyvale,CA, USA) and normalized to Renilla luciferase activity.

### Chromatin immunoprecipitation assay

ChIP assay was performed using a ChIP assay kit (Abcam, Cambridge, UK) according to the manufacturer’s protocol. Briefly, cell lysates were incubated with anti-STAT1 antibody (1:500, CST, 14994) or IgG (1:500, Abcam, ab172730). The DNA–protein cross-linked complexes were precipitated, and the purified DNA was analyzed by RT-PCR using the SYBR Green PCR Master Mix (ThermoFisher, CA, USA). Primer sequences are shown in Supplementary Data 4.

### Statistical analysis

All results are expressed as mean ± standard deviation. Student’s unpaired t tests and two-way analysis of variance (ANOVA) followed by Dunnett’s test were used to analyze data. A *p*-value of less than 0.05 was considered to be statistically significant. All statistical analyses were done with the SPSS 14.0 software.

### Ethics approval and consent to participate

All study surgical procedures and experiment protocols were performed in accordance with standard guidelines approved by the Ethics Committee of Experimental Research, Shanghai Medical College, Fudan University (Shanghai, China).

## Supplementary information

Supplementary Figure

Supplementary Table 1

Supplementary Data 1

Supplementary Table 2

Supplementary Data 2

Supplementary Data 3

Supplementary Data 4
