# Supplementary figures and images for: TLR4 promotes microglial pyroptosis via lncRNA-F630028O10Rik by activating PI3K/AKT pathway after spinal cord injury
Source: Cell Death Dis. 2020 Aug 10;11(8):693. doi: 10.1038/s41419-020-02824-z (PMC7443136; doi:10.1038/s41419-020-02824-z)

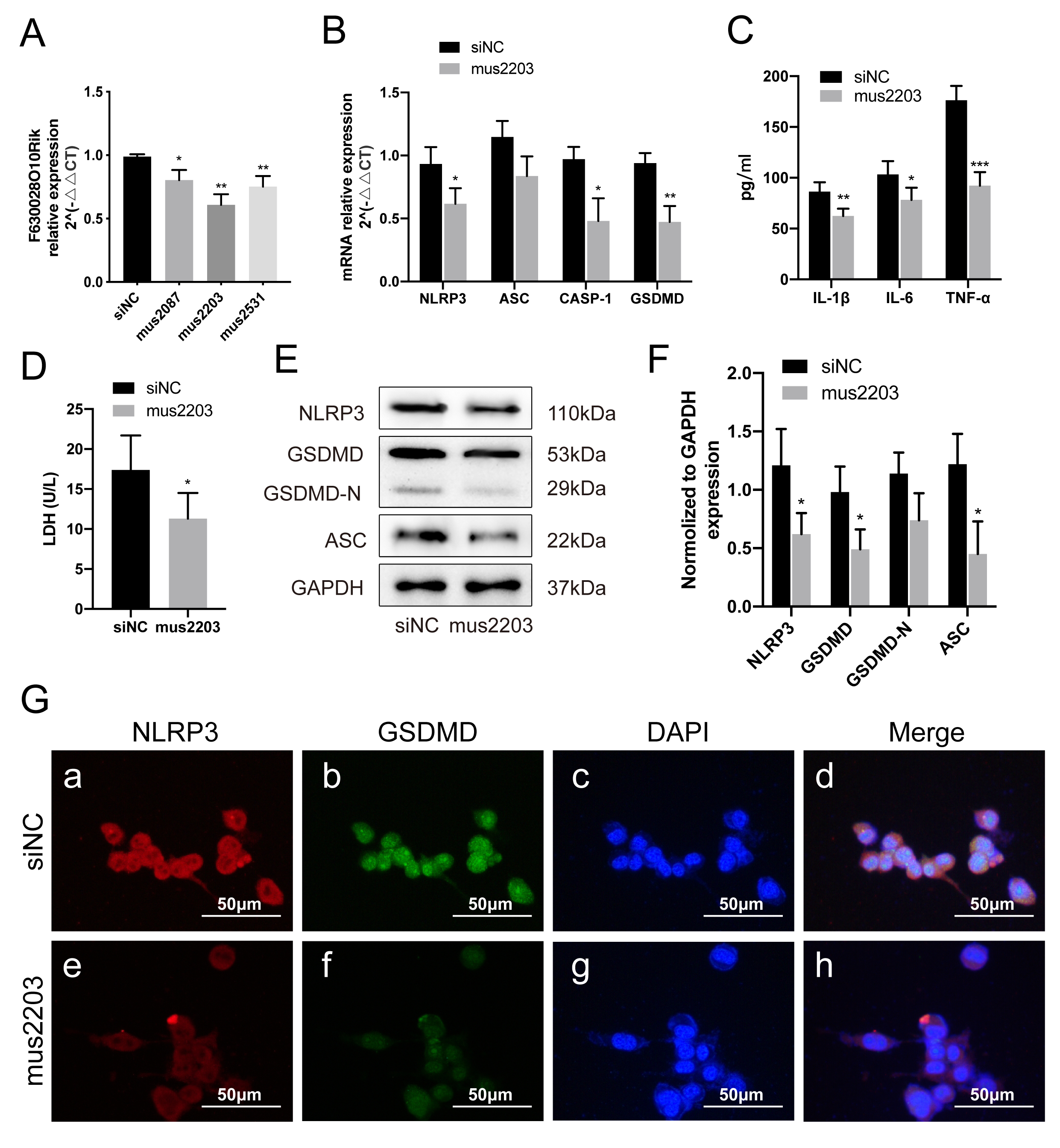

Supplement: Supplementary file 1 — Supplementary Figure [file 41419_2020_2824_MOESM1_ESM.tif]
