# Supplementary material for: TLR4 promotes microglial pyroptosis via lncRNA-F630028O10Rik by activating PI3K/AKT pathway after spinal cord injury
Source: Cell Death Dis. 2020 Aug 10;11(8):693. doi: 10.1038/s41419-020-02824-z (PMC7443136; doi:10.1038/s41419-020-02824-z)
Supplement: Supplementary file 2 — Supplementary Table 1 [file 41419_2020_2824_MOESM2_ESM.docx]

Table 1. Clinical characteristics of patient samples

|  | **Total** | **With SCI** | **Without SCI** | **P value** |
| --- | --- | --- | --- | --- |
| **Patients(n)** | 40 | 20 | 20 |  |
| **Ages(years)** | 49.4±10.4 | 49.7±10.4 | 49.2±10.8 | 0.87 |
| **Weight(kg)** | 67.2±7.8 | 66.9±7.6 | 67.6±8.1 | 0.77 |
| **LncRNA**  **F630028O10Rik** | 4.7±3.4 | 7.0±3.2 | 2.3±1.2 | ＜0.0001 |
| **Sex(male=1)** |  |  |  |  |
| Male | 25 | 14 | 11 | 0.51 |
| Female | 15 | 6 | 9 |  |
