# Supplementary material for: TLR4 promotes microglial pyroptosis via lncRNA-F630028O10Rik by activating PI3K/AKT pathway after spinal cord injury
Source: Cell Death Dis. 2020 Aug 10;11(8):693. doi: 10.1038/s41419-020-02824-z (PMC7443136; doi:10.1038/s41419-020-02824-z)
Supplement: Supplementary file 3 — Supplementary Data 1 [file 41419_2020_2824_MOESM3_ESM.docx]

**1.Sequence of siRNAs target lncRNAF630028010Rik**

| Genes | Primer sequences (5’-3’) |
| --- | --- |
| F630028010Rik-mus-2531 | CCUCUUGGCACUAUUGAAATT  UUUCAAUAGUGCCAAGAGGTT |
| F630028010Rik-mus-2203 | GGGUCUACAAGUCUGGUAUTT  AUACCAGACUUGUAGACCCTT |
| F630028010Rik-mus-2087 | CUGCUGCCUACAUAUGAAUTT  AUUCAUAUGUAGGCAGCAGTT |
| NC | UUCUCCGAACGUGUCACGUTT  ACGUGACACGUUCGGAGAATT |

**2. Construction of Lentiviral Interference Vector**

| Gene | F630028O10Rik-2203 | | Target | F630028O10Rik-2203 |
| --- | --- | --- | --- | --- |
| Vector | LV3(H1/GFP&Puro)-F630028O10Rik-2203 | | | |
| Target sequence | GGGTCTACAAGTCTGGTATGC | | | |
| shDNA  template  sequence | S | 5'-GATCCGGGTCTACAAGTCTGGTATGCTTCAAGAGAGCATACCAGACTTGTAGACCCTTTTTTG-3' | | |
|  | A | 5'-AATTCAAAAAAGGGTCTACAAGTCTGGTATGCTCTCTTGAAGCATACCAGACTTGTAGACCCG-3' | | |
|  | 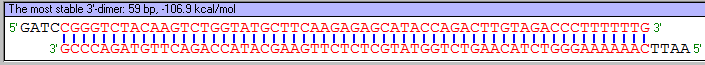 | | | |
| Transcript  sequence  structure | GGGTCTACAAGTCTGGTATGCTTCAAGAGAGCATACCAGACTTGTAGACCCTT | | | |
|  | 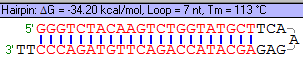 | | | |
| Sequencing results | CCCCTATTCTTTCTTTGCAGTTATAAATACTGAATAATAAGATGACATGAACTACTACTGCTAGAGATTTTCCACACTGACTGAAAGGGTCTGAGGGATCTCTAGTTACCAGAGTCACACAACAGACGGGCACACACTACTTGAAGCACTCAAGGCAAGCTTTATTGAGGCTTAAGCAGTGGGTTCCCTAGTTAGCCAGAGAGCTCCCAGGCTCAGATCTGGTCTAACCAGAGAGACCCAGTAGAAGCAAAAAGCAGAATCGAAGAATTCAAAAAAGGGTCTACAAGTCTGGTATGCTCTCTTGAAGCATACCAGACTTGTAGACCCGGATCCAAGTGGTCTCATACAGAACTTATAAGATTCCCAAATCCAAAGACATTTCACGTTTATGGTGATTTCCCAGAACACATAGCGACATGCAAATATTGCAGGGCGCCACTCCCCTGTCCCTCACAGCCATCTTCCTGCCAGGGCGCACGCGCGCTGGGTGTTCCCGCCTAGTGACACTGGGCCCGCGATTCCTTGGAGCGGGTTGATGACGTCAGCGTTCCAATTCTTGACATCGTTGGGAGTGAATTAGCCCTTCCAGTCCCCCCTTTTCTTTTAAAAAGTGGCTAAGATCTACAGCTGCCTTGTAAGTCATTGGTCTTAAAGGTACCAGGCGGGGAGGCGGCCCAAAGGGAGATCCGACTCGTCTGAGGGCGAAGGCGGAGACGCGGAAGAGGCCGCAGAGCCGGCAGCAGGCCGCGGGAAGGAAGGTCCGCTGGATTGAGGGCCGAAGGGACGTAGCAGAAGGACGTCCCGCGCAGAATCCAGGTGGCAACACAGGCGAGCAGCCAAGGAAAGGACGATGATTTCCCCGACAACACCACGGAATTGTCAGTGCCCAACAGCCGAGCCCCTGTCCAGCAGCGGGCAAGGCAGGCGGCGATGAATTCCGCCGTGGCAATAGGGAGGGGGAAAACGAAAATCCCGGAAAGAACTTACAA | | | |
| Vector structure | 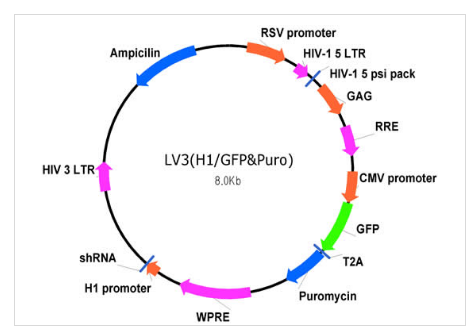 | | | |

**3. Construction of Lentiviral Overexpression Vector**

| Gene | F630028O10Rik |
| --- | --- |
| Method | The F630028O10Rik gene sequence of mouse origin was synthesized, and EcoRI-XbaI restriction sites (Red) were added at both ends. The resulting gene fragment was blunt-ended cloned into the PUC57 vector. |
| F630028O10Rik  sequence | GGATCCTTTCTGTGGCCTCTATAGTTCGGCTCAGCACCTATATCCCATCCAAGACAAAGGCACCACTTCAATGACAGGTTACCAGTCTTTGGTTGGGAAAGAAGATGAGACTGCTGCTTGAATGTACACCTCTAGGCAGAGCTGAACATCCTTTAGAAAACTGCTGGTGGTCTGAAAGGTGTGAACTGGAACATCAAGAATGAAAATGAAATAGTCCCTTACCCAGCTTAAGGACTGACTTCTCCATCAATCCTGGAAAACAGTATTTATAATAATCTATTAAGTCAGTGCACTAGAACTCTTTAGCCCCTTTCATGGCTCTTTTCCAGTGTTCTTGGGTTATGTGAGGTCGGATAGGGCAATTGAGAGGCTATGGATATCTCTAAAGTGTCTAGCTCTCCTTACAGCAGATGATAAGCAGAGATGCTGTCAAGAAAACCAAAACCAATAACCCATTACTCCTTTCTCTTTTAATCACTTTCCTTGGCTTTTACTAGCTCCAGAGTTGGAAATAAGGTCTCTTTAAGCTCCCTTCTAAAAAGCTCTAGAAGTCTCATCCAACATTTTTCAAGACCTTGAGATGTAAGATTTCTGTCTCTATTTCTAGAGAAGACAATTGATGCCCAAGAAAACTTTAAACCTGGGTAGGACAAAATTTGATACTATCAAGCATTTTGAATAACAGGATCATGATGCTAAACAAAATATCAAATAAATTGAGATGGCTTGGCTCTTCTGGCAGCTGCCCTGGTAGCTATGAGTTCAAACATCTTAATACTTCCCTGCAAAATCCACTGCCTGTTTTGCTCTTTGTTGCTGTTGCTCTCCCTTTTGCAAGGGAATATAAAAAGCAGACAAGAAAAAGTGGTGGGGTGGACAGCCAAGGGACACAATGAGCTGATGGATGATATAATTCCCAAACTGTAAACAACTTGTTTTATGACCTTTTGGAAGTCTTTCCCTAAACATCAGACTTCATTTTCTCATTTATAATCTTGAATGGGTAAAACTAGATGACTTATAAGGGCATTTTAATATCATTAATTCTTTACTGCTTATGAGAAAAGGCAACCAAGTTAGACAAAGCCGGCTAAGGCCACAAGAAATTACTGTTTCAATTCTGTTAAATAAATTATCTATCTTTTCCCCTGTTAGTATCTATCTTATATGTTTAATTTTAAAGCACATTTTAGTCACTTTTATTTATGTACAGGGGATCAAATACAGAGTATTGTACAAGTTAGGTAAGTGTTCTGCTACTGAGTTTTATCTCTAGATCTTGATTGTTTACTTTTTTGTTTGAGAAAGGGTAGTATTATATAGCCCAAATTGACTTTAAATCTGAGGTTCTCCTATCTCTGTCTATGAAAGCTTGTATGCACACATGTACTATCATACCTGGCTATAGCCACAATTTTTGAGTATTTGTTAGGTAACAAACACTAAATTAATGGCTTTATATGCATTATTACATATTCTTGCCATGATTTCATGGGCATTTTTGTGGCTATGGAAGAAGTAGGTTTCAGGGACATAAACTCATTTATTTAAGGTGTTCAGCTAGTTAATGGTAGAGGTAGAATTCAAATCCAGTTTTAAAAATCAACATCTAGGTACATAACCAGGGTAAACAGAGCATACAAGAAGTTGATAGGAGGAAGGAACACATAAATTGTCCCCGAGGACAGAATGATGACAATTTTTTCCTGTTTCAGCTGAATAGAAGAGTTAATTGAGGCTTGGGCCACCAGAATCTCCAGACAAACACCCAGGATCCCCGTTTTTGTTTGGAGCATTAGCGTGTTTTGTTCACTTCCCTCTTTTGGTGTGTCTTAGTCAGTCATGTTAGTGTCTGCCATTTGTCTCACACTTAGGGTAAGATATAATCACCCTATTTTTTTCTCTTTCCAGTTGCACATCTTCCAGCATGTTCTTGCTGCCCAGTGGAGGTTCCTGATCTGGCCATCTGCAGTGTCACGCTCCGTGTATTTGACAAGCTGAGTTGGACACTCTGTGTGGTAGAGTGTCAGTTTGTCAAATACCCCAAGTGTGGCTCATGCCTATCAGCTCCAGGTCCAGGACAGAGCACATAGCCTGCTGCCTACATATGAATGCTTATGAAACATGAAGCTCTCTGGTGTTATTCTATGTCTTTAAGGGAACTATAACATTTATTCAGAGAACATCAGAGTAGGAACGATGCAGCTCTGGGGTCTACAAGTCTGGTATGCATCTGTGTACATTGTGAAAATGCCATGGACTGCATAGTGGTACATGAGGGATACAGGGAATGAGAGTATTATTTGATTTATTATTAATGTCTTCTTTCACTTTCCTCTTCTTGCAGCCAGAATAGCAGAATTTTCATACAAGCATCATGAATTCTATGAGAAAGTAGGAAATAAATATCCACTGTCTGGCCTTCGGAGCACATCTTAAGCTACCTACATTTTCTCTTTTCCACTTTGCTTTTATCATTCTTAAAAGAACCTTCTTGTTTATCTTCATTGGGCTTTCAGACACTACCAAATTCCAATTCCTCTTGGCACTATTGAAAGTTAACCTATACTTTTCTTTACTCCTTGGCTAACCACATACTGGTATGTGCTATTATATGTCCTACCATCCAACACATCTTCTAAAGTTGTCAGTGAATTTTTCCAGGGTATGTTCTTGAATTCCCTATTATATTGTAAGAATCTAGAGCTTATTATATTCAGTTGATTGCTGTCTCTCATACCATTGTCCAACATTTTAATTATTAATAAATATTTACCAATTAAAAAAAAAAAAAAAAAAAAATCTAGA |
| Vector structure | 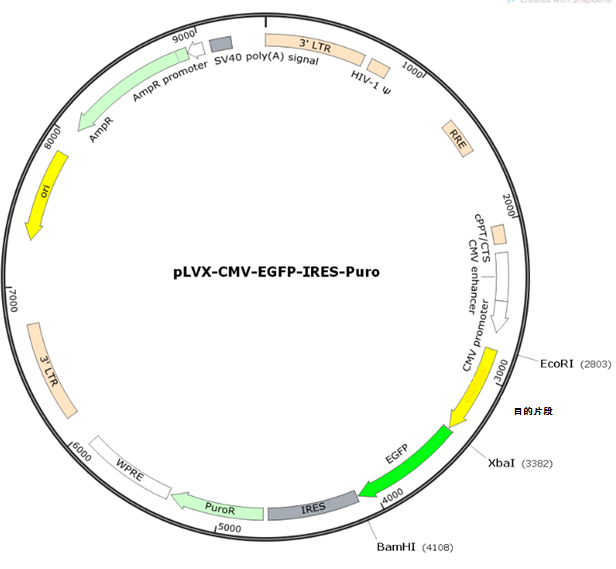 |

**4.pCD-col1a1 sequence:**

GCTAGCATGTTCAGCTTTGTGGACCTCCGGCTCCTGCTCCTCTTAGGGGCCACTGCCCTCCTGACGCATGGCCAAGAAGACATCCCTGAAGTCAGCTGCATACACAATGGCCTAAGGGTCCCCAATGGTGAGACGTGGAAACCCGAGGTATGCTTGATCTGTATCTGCCACAATGGCACGGCTGTGTGCGATGACGTGCAATGCAATGAAGAACTGGACTGTCCCAACCCCCAAAGACGGGAGGGCGAGTGCTGTGCTTTCTGCCCGGAAGAATACGTATCACCAAACTCAGAAGATGTAGGAGTCGAGGGACCCAAGGGAGACCCTGGCCCCCAAGGCCCAAGGGGACCCGTTGGCCCCCCTGGACGAGATGGCATCCCTGGACAGCCTGGACTTCCTGGTCCTCCTGGTCCCCCTGGGCCCCCCGGACCCCCTGGCCTTGGAGGAAACTTTGCTTCCCAGATGTCCTATGGCTATGATGAAAAATCAGCTGGAGTTTCCGTGCCTGGCCCCATGGGTCCTTCTGGTCCTCGTGGTCTCCCTGGCCCCCCTGGTGCACCTGGTCCACAAGGTTTCCAAGGCCCCCCTGGTGAACCTGGCGAGCCTGGCGGTTCAGGTCCAATGGGTCCCCGAGGTCCCCCTGGCCCTCCTGGCAAGAATGGAGATGATGGGGAAGCTGGCAAGCCCGGCCGTCCTGGTGAGCGTGGACCTCCTGGACCTCAGGGTGCTCGTGGATTGCCTGGAACAGCTGGCCTCCCTGGAATGAAGGGACACCGAGGCTTCAGTGGTTTGGATGGTGCCAAAGGAGATGCTGGTCCTGCTGGTCCTAAGGGAGAGCCCGGCAGTCCTGGTGAAAACGGAGCTCCTGGCCAGATGGGTCCCCGAGGTCTGCCCGGTGAGAGAGGTCGCCCTGGACCTCCTGGCACTGCTGGTGCTCGCGGTAACGATGGTGCTGTTGGTGCTGCTGGACCCCCTGGTCCCACCGGCCCCACTGGCCCTCCTGGCTTCCCTGGTGCAGTTGGTGCTAAGGGTGAAGCTGGTCCCCAAGGAGCTAGAGGCTCTGAAGGTCCCCAGGGTGTGCGTGGTGAGCCCGGACCCCCTGGCCCTGCTGGTGCTGCCGGCCCTGCTGGAAACCCTGGTGCTGATGGACAACCTGGCGCTAAAGGTGCCAATGGTGCTCCTGGTATTGCTGGTGCTCCTGGCTTCCCTGGTGCCCGAGGCCCCTCTGGACCCCAGGGCCCCAGCGGCCCTCCAGGTCCCAAGGGTAACAGTGGTGAACCTGGTGCTCCTGGCAACAAAGGAGACACTGGTGCCAAAGGAGAACCCGGTGCTACTGGAGTTCAAGGTCCCCCAGGCCCTGCCGGAGAAGAAGGAAAACGAGGAGCCCGTGGTGAGCCTGGACCTTCCGGACTGCCTGGACCTCCTGGCGAGCGTGGTGGACCTGGTAGCCGTGGTTTCCCTGGTGCTGATGGTGTTGCTGGCCCCAAGGGTCCTTCCGGTGAACGTGGTGCTCCCGGACCTGCTGGTCCCAAAGGTTCTCCTGGTGAAGCTGGTCGCCCCGGTGAAGCTGGTCTCCCTGGTGCCAAGGGTCTCACTGGCAGTCCTGGCAGCCCTGGTCCTGATGGCAAAACCGGCCCCCCTGGTCCCGCTGGTCAAGATGGTCGCCCTGGACCCGCAGGTCCTCCTGGAGCCCGTGGCCAGGCTGGTGTGATGGGATTCCCTGGACCTAAGGGTACCGCTGGAGAACCTGGAAAGGCTGGAGAGCGAGGCCTTCCCGGACCCCCTGGCGCTGTTGGTCCTGCTGGCAAAGATGGAGAAGCTGGAGCTCAGGGAGCCCCTGGCCCTGCTGGTCCTGCTGGTGAGAGAGGTGAACAAGGTCCCGCTGGCTCCCCTGGATTCCAGGGTCTTCCTGGTCCTGCCGGTCCTCCTGGTGAAGCAGGCAAGCCTGGTGAACAGGGTGTTCCTGGAGACCTTGGTGCCCCTGGACCCTCTGGCGCAAGAGGCGAGAGAGGTTTCCCTGGTGAACGTGGTGTACAAGGTCCCCCAGGTCCTGCTGGTCCCCGAGGAAACAATGGTGCCCCCGGCAACGATGGTGCCAAGGGTGATACTGGTGCCCCCGGAGCTCCCGGTAGCCAGGGTGCCCCCGGTCTTCAGGGAATGCCTGGTGAACGTGGTGCAGCTGGTCTTCCAGGTCCTAAGGGTGACAGAGGTGATGCTGGTCCCAAAGGTGCTGATGGTTCTCCTGGTAAAGATGGTGCCCGTGGTCTGACTGGTCCCATTGGTCCTCCTGGCCCTGCTGGTGCCCCTGGTGACAAGGGTGAAGCTGGTCCCAGTGGTCCTCCCGGTCCCACCGGAGCCCGTGGTGCTCCCGGAGACCGTGGTGAGGCTGGTCCCCCTGGTCCTGCTGGCTTTGCCGGCCCCCCTGGTGCTGATGGCCAACCTGGTGCGAAAGGTGAACCTGGTGATACTGGTGTTAAAGGTGATGCTGGTCCTCCTGGCCCTGCTGGTCCTGCTGGACCCCCCGGCCCCATTGGTAACGTTGGTGCTCCTGGACCCAAAGGTCCTCGTGGTGCTGCTGGTCCCCCTGGTGCTACTGGCTTCCCTGGTGCTGCTGGCCGTGTCGGTCCCCCTGGTCCCTCTGGAAATGCTGGACCCCCTGGCCCTCCCGGTCCCGTTGGCAAAGAAGGGGGCAAAGGTCCCCGTGGTGAGACTGGCCCTGCTGGACGTCCTGGTGAAGTTGGTCCCCCAGGTCCCCCCGGTCCTGCTGGTGAGAAAGGATCTCCTGGTGCTGATGGACCTGCTGGCTCTCCTGGTACCCCTGGACCTCAGGGTATTGCTGGACAACGTGGTGTGGTCGGTCTTCCCGGTCAGAGAGGAGAAAGAGGCTTCCCTGGTCTTCCTGGCCCCTCTGGTGAACCTGGCAAACAAGGTCCTTCTGGATCAAGTGGTGAACGCGGTCCCCCTGGCCCCATGGGGCCCCCTGGATTGGCTGGTCCCCCTGGTGAATCTGGACGTGAGGGATCCCCTGGTGCTGAAGGCTCCCCTGGAAGGGATGGTGCTCCCGGGGCCAAGGGTGACCGTGGTGAGACTGGCCCCGCTGGCCCCCCTGGTGCCCCTGGTGCTCCCGGTGCTCCCGGCCCTGTTGGTCCCGCTGGCAAGAATGGCGATCGTGGTGAGACTGGTCCTGCTGGTCCTGCTGGTCCCATTGGCCCTGCTGGTGCCCGTGGCCCTGCTGGACCCCAAGGCCCCCGTGGTGACAAGGGTGAGACAGGCGAACAAGGTGACAGAGGCATAAAGGGTCATCGTGGCTTCTCTGGTCTCCAGGGTCCTCCTGGTTCTCCTGGTTCTCCTGGTGAACAAGGCCCCTCTGGAGCTTCAGGTCCTGCAGGCCCCCGGGGTCCCCCTGGCTCTGCTGGTTCTCCTGGCAAAGACGGACTCAACGGTCTCCCTGGCCCCATTGGTCCCCCTGGTCCTCGAGGTCGCACTGGTGACAGCGGCCCTGCTGGTCCCCCCGGCCCTCCTGGACCCCCTGGCCCTCCTGGACCTCCCAGTGGCGGTTATGACTTCAGCTTCCTGCCTCAGCCACCTCAAGAGAAGTCTCAAGATGGTGGCCGCTACTACCGGGCCGATGATGCTAACGTGGTTCGTGACCGTGACCTTGAGGTGGACACCACCCTCAAGAGCCTGAGTCAGCAGATTGAGAACATCCGCAGCCCCGAAGGCAGCCGCAAGAACCCTGCCCGCACATGCCGCGACCTCAAGATGTGCCACTCTGACTGGAAGAGCGGAGAGTACTGGATCGACCCTAACCAAGGCTGCAACCTGGACGCCATCAAGGTCTACTGCAACATGGAGACAGGTCAGACCTGTGTGTTCCCTACTCAGCCGTCTGTGCCTCAGAAGAACTGGTACATCAGCCCGAACCCCAAGGAAAAGAAGCACGTCTGGTTTGGAGAGAGCATGACCGATGGATTCCCGTTCGAGTACGGAAGCGAGGGCTCCGACCCCGCCGATGTCGCTATCCAGCTGACCTTCCTGCGCCTAATGTCCACCGAGGCCTCCCAGAACATCACCTATCACTGCAAGAACAGCGTAGCCTACATGGACCAGCAGACTGGCAACCTCAAGAAGGCCCTGCTCCTCCAGGGATCCAACGAGATCGAGCTCAGAGGCGAAGGCAACAGTCGCTTCACCTACAGCACCCTTGTGGACGGCTGCACGAGTCACACCGGAACTTGGGGCAAGACAGTCATCGAATACAAAACCACCAAGACCTCCCGCCTGCCCATCATCGATGTGGCTCCCTTGGACATTGGTGCCCCAGACCAGGAATTCGGACTAGACATTGGCCCTGCCTGCTTCGTGTAAGCGGCCGC
