# Supplementary material for: TLR4 promotes microglial pyroptosis via lncRNA-F630028O10Rik by activating PI3K/AKT pathway after spinal cord injury
Source: Cell Death Dis. 2020 Aug 10;11(8):693. doi: 10.1038/s41419-020-02824-z (PMC7443136; doi:10.1038/s41419-020-02824-z)
Supplement: Supplementary file 4 — Supplementary Table 2 [file 41419_2020_2824_MOESM4_ESM.docx]

**Table 1. Primer sequence.**

| Genes | Primer sequence (5’-3’) |
| --- | --- |
| TLR4 | Forward：ATGGCATGGCTTACACCACC  Reverse：GAGGCCAATTTTGTCTCCACA |
| GAPDH | Forward：AGGTCGGTGTGAACGGATTTG  Reverse：TGTAGACCATGTAGTTGAGGTCA |
| NLRP3 | Forward：ATTACCCGCCCGAGAAAGG  Reverse：TCGCAGCAAAGATCCACACAG |
| GSDMD | Forward：CCATCGGCCTTTGAGAAAGTG  Reverse：ACACATGAATAACGGGGTTTCC |
| ASC | Forward：CTTGTCAGGGGATGAACTCAAAA  Reverse：GCCATACGACTCCAGATAGTAGC |
| 2900052N01Rik | Forward：GAGAGGAAAGTATTCAGGGGCAC  Reverse：ATGGAGAAGTGGAAAACAGGAAGT |
| 1500011B03Rik | Forward：GCCTGGTGCTGATGGGAAC  Reverse：GCCCAGTTCTGATTCCACCA |
| Gm15521 | Forward：TGTGCAGAACAACAGTTTGGTTC  Reverse：TCAGTGAGAGAAGCCAATGCAG |
| Neat1 | Forward：CTCAGACCCTCAGTTTCCCACC  Reverse：ATGCCATCCAGGTTCCAAGG |
| Xist | Forward：CTCAGACCCTCAGTTTCCCACC  Reverse：ATGCCATCCAGGTTCCAAGG |
| Sox2ot | Forward：TACCGAGAAGCAAACCTGACA  Reverse：AAGCACGGAGAATCCATTTAGG |
| F630028O10Rik | Forward：AAGACAAAGGCACCACTTCAATG  Reverse：CCACCAGCAGTTTTCTAAAGGATG |
| TNF-α | Forward：CCCTCACACTCAGATCATCTTCT  Reverse：GCTACGACGTGGGCTACAG |
| IL-1β | Forward：CGAAGACTACAGTTCTGCCATT  Reverse：GACGTTTCAGAGGTTCTCAGAG |
| IL-6 | Forward：TAGTCCTTCCTACCCCAATTTCC  Reverse：TTGGTCCTTAGCCACTCCTTC |
| Caspase-1 | Forward：ACAAGGCACGGGACCTATG  Reverse：TCCCAGTCAGTCCTGGAAATG |
| U6 | Forward：CGATACAGAGAAGATTAGCATGGC  Reverse：AACGCTTCACGAATTTGCGT |
| Col1a1 | Forward：CGGTGCTACTGGAGTTCAAGGT  Reverse：GCTACCAGGTCCACCACGCT |
| Sgk1 | Forward：GAGCTTATGAACGCTAACCCCT  Reverse：AGAAGAACCTTTCCAAAACTGCC |
| Tnr | Forward：GATGGAAGCCGCAAAGAGC  Reverse：GAGGTGAGACTGCTCCTTGTGG |
| Kdr | Forward：CACTCTCCACCTTCAAAGTCTCAT  Reverse：GTGCTCAAAAACATCTTCGCC |
| Col5a1 | Forward：CCAGGTCCCAAAGGTGCTAA  Reverse：CATCAATGTTCCGCCGAGT |
| Hras | Forward：TCTGGCTGGAAGTAGGAGGTG  Reverse：AACACAAGACAGTTGGGACAGG |
| STAT1 | Forward：TCACAGTGGTTCGAGCTTCAG  Reverse：CGAGACATCATAGGCAGCGTG |
