# Supplementary material for: TLR4 promotes microglial pyroptosis via lncRNA-F630028O10Rik by activating PI3K/AKT pathway after spinal cord injury
Source: Cell Death Dis. 2020 Aug 10;11(8):693. doi: 10.1038/s41419-020-02824-z (PMC7443136; doi:10.1038/s41419-020-02824-z)
Supplement: Supplementary file 6 — Supplementary Data 3 [file 41419_2020_2824_MOESM6_ESM.docx]

**1. Col1a1-mmu-miR-1231-5p sequence：**

CTCGAGGTTTTTTTTCCTGAAGGTGCTATTTAACAAGGGAGAAGAGAGTGCGGGGACTTCACCCTGCCCACTCTCTACTCTCTCTCCACTCTTCTAGTTCCTGGGCCTATCTGATCTCTCTCTTTCTTCTGAAACCCTCCCCTCTTGCTGCTGCTCCCTCCCCTCTGCCTCTCTCTTGGTCTGTCCTGCATCAGGGTTTCAGAGCACCACTTTCCAAAGCACAAAACAGTTTTTACCCCTGGGCTGGGAGGAAACAAGAGACTCTGTACCTATTTTGTATGTGTATAATAATTTGAGATGTTTTTAATTATTTTGATTGCTGGAATAAAGCATGTGGAAATGACCCGCGGCCGC

**2. Col1a1-mmu-miR-1231-5p-mut sequence：**

CTCGAGGTTTTTTTTCCTGAAGGTGCTATTTAACAAGGGAGAAGAGAGTGCGGGGACTTCACCCTGCCCACTCTCTACTCTCTCTCCACTCTTCTAGTTCCTGGGCCTATCTGATCTCTCTCTTTCTTCTGAAACCCTCCCCTCTTGCTGCTGCTCCCTCCCCTCTGCCGATGCCATCGTCTGTCGAGTAGTACAGTTTCAGAGCACCACTTTCCAAAGCACAAAACAGTTTTTACCCCTGGGCTGGGAGGAAACAAGAGACTCTGTACCTATTTTGTATGTGTATAATAATTTGAGATGTTTTTAATTATTTTGATTGCTGGAATAAAGCATGTGGAAATGACCCGCGGCCGC

**3. F630028O10Rik-mmu-miR-1231-5p sequence：**

CTCGAGGAAGTCTCATCCAACATTTTTCAAGACCTTGAGATGTAAGATTTCTGTCTCTATTTCTAGAGAAGACAATTGATGCCCAAGAAAACTTTAAACCTGGGTAGGACAAAATTTGATACTATCAAGCATTTTGAATAACAGGATCATGATGCTAAACAAAATATCAAATAAATTGAGATGGCTTGGCTCTTCTGGCAGCTGCCCTGGTAGCTATGAGTTCAAACATCTTAATACTTCCCTGCAAAATCCACTGCCTGTTTTGCTCTTTGTTGCTGTTGCTCTCCCTTTTGCAAGGGAATATAAAAAGCAGACAAGAAAAAGTGGTGGGGTGGACAGCCAAGGGACACAATGAGCTGATGGATGATATAATTCCCAAACTGTAAACAACTTGTTTTGCGGCCGC

**2. F630028O10Rik-mmu-miR-1231-5p-mut sequence：**

CTCGAGGAAGTCTCATCCAACATTTTTCAAGACCTTGAGATGTAAGATTTCTGTCTCTATTTCTAGAGAAGACAATTGATGCCCAAGAAAACTTTAAACCTGGGTAGGACAAAATTTGATACTATCAAGCATTTTGAATAACAGGATCATGATGCTAAACAAAATATCAAATAAATTGAGATGGCTTGGAGCGTACGTGATCATGCATGGTAGCTATGAGTTCAAACATCTTAATACTTCCCTGCAAAATCCACTGCCTGTTTTGCTCTTTGTTGCTGTTGCTCTCCCTTTTGCAAGGGAATATAAAAAGCAGACAAGAAAAAGTGGTGGGGTGGACAGCCAAGGGACACAATGAGCTGATGGATGATATAATTCCCAAACTGTAAACAACTTGTTTTGCGGCCGC
