# Supplementary material for: TLR4 promotes microglial pyroptosis via lncRNA-F630028O10Rik by activating PI3K/AKT pathway after spinal cord injury
Source: Cell Death Dis. 2020 Aug 10;11(8):693. doi: 10.1038/s41419-020-02824-z (PMC7443136; doi:10.1038/s41419-020-02824-z)
Supplement: Supplementary file 7 — Supplementary Data 4 [file 41419_2020_2824_MOESM7_ESM.docx]

| **Matrix ID** | **Name** | **Score** | **Relative score** | **Sequence ID** | **Start** | **End** | **Strand** | **Predicted sequence** |
| --- | --- | --- | --- | --- | --- | --- | --- | --- |
| [**MA0517.1**](http://jaspar.genereg.net/matrix/MA0517.1) | STAT1::STAT2 | 11.339 | 0.847363509414 | mm10_knownGene_ENSMUST00000133125.2 | 313 | 327 | + | ttagttccctttctc |
| [**MA0517.1**](http://jaspar.genereg.net/matrix/MA0517.1) | STAT1::STAT2 | 9.63747 | 0.823049622144 | mm10_knownGene_ENSMUST00000133125.2 | 374 | 388 | + | ttagttacccttttt |
| [**MA0517.1**](http://jaspar.genereg.net/matrix/MA0517.1) | STAT1::STAT2 | 8.42199 | 0.8056813684 | mm10_knownGene_ENSMUST00000133125.2 | 1929 | 1943 | + | tctgtttcctcttca |

1. **JASPAR prediction results：**

**2. Primer sequence：**

| Genes | Primer sequence (5’-3’) |
| --- | --- |
| F630028O10Rik-P1 | Forward：CAGGGATTATGAACACGAAGATTT  Reverse：TCCTTAAGTCAATGTGACCTTCAAG |
| F630028O10Rik-P2 | Forward：CTTGAAGGTCACATTGACTTAAGGA  Reverse：CAGGAACTAATGGTGAAGACAGAAT |
| F630028O10Rik-P3 | Forward：GCATTAGCAACTACTCCTTTCACA  Reverse：GGAAGGTCACATGTTCTGAAGGA |
